# A step-by-step guide to performing cancer metabolism research using custom-made media

**DOI:** 10.26508/lsa.202503529

**Published:** 2025-12-10

**Authors:** Sophie Seifert, Francisco Yanqui-Rivera, Tim Kühn, Lena Elise Høyland, Toman Borteçen, Bella Agranovich, Lara Elea Eckhardt, Martin Herdt, Alessa Henneberg, Verena Panitz, Faisal Hayat, Marie Migaud, Gernot Poschet, Ifat Abramovich, Eyal Gottlieb, Jeroen Krijgsveld, Mathias Ziegler, Mirja Tamara Prentzell, Christiane A Opitz

**Affiliations:** 1 German Cancer Research Center (DKFZ), Division of Metabolic Crosstalk in Cancer and The German Cancer Consortium (DKTK), DKFZ Core Center Heidelberg, Heidelberg, Germany; 2 Faculty of Biosciences, Heidelberg University, Heidelberg, Germany; 3 Department of Biomedicine, University of Bergen, Bergen, Norway; 4 German Cancer Research Center (DKFZ), Division of Proteomics of Stem Cells and Cancer, Heidelberg, Germany; 5 Medical Faculty, Heidelberg University, Heidelberg, Germany; 6 Ruth and Bruce Rappaport Faculty of Medicine, Technion—Israel Institute of Technology, Haifa, Israel; 7 Department of Neurology and National Center for Tumor Diseases, Heidelberg University Hospital, Heidelberg, Germany; 8 Mitchell Cancer Institute, University of South Alabama, Mobile, AL, USA; 9 Centre for Organismal Studies (COS), Heidelberg University, Heidelberg, Germany; 10 Department of Cancer Biology, University of Texas MD Anderson Cancer Center, Houston, TX, USA

## Abstract

The preparation of custom-made media offers precise control over nutrient composition, enabling detailed studies of cellular metabolism. We demonstrate how self-made media formulations enable diverse assay designs and readouts to assess cancer metabolism. Self-made media can be used in Seahorse assays to measure mitochondrial respiration under defined conditions. In nutrient deprivation experiments, amino acid or vitamin removal can uncover how cancer cells adapt to metabolic stress. Using labeled amino acids enables analysis of nascent protein synthesis and translational regulation, while stable-isotope tracing reveals metabolic fluxes through key pathways. This guide presents a suite of metabolic assays using custom-made media, covering experimental design, the selection of controls, sample preparation, data acquisition, and interpretation. The accompanying online media calculator “Media Minds” streamlines the creation of custom media formulations, ensuring accuracy and reproducibility.

## Abstract (precis)

We present a detailed step-by-step protocol for preparing custom-made cell culture media and provide guidance on its use in cancer metabolism experiments.

## Graphical Abstract

**Figure:**
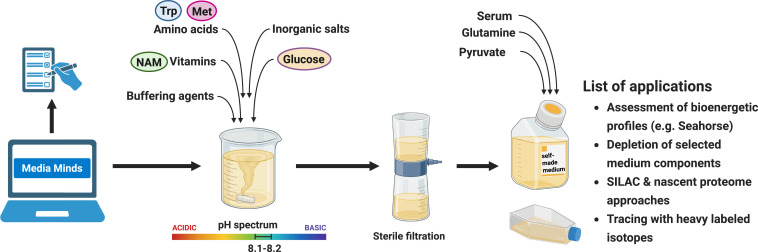

## Introduction

Metabolism is fundamental to the biology of cancer cells, driving not only their rapid proliferation but also their ability to survive under the stressful conditions often present in tumors. Cancer cells reprogram their metabolism to quickly generate ATP and to produce the building blocks necessary for cell proliferation (Hanahan & Weinberg, 2000, 2011; Pavlova et al, 2022). However, the importance of metabolism in cancer extends beyond its traditional roles in sustaining cancer cell proliferation. Metabolites exert important signaling functions that regulate key aspects of cancer biology, including cell survival and motility as well as immune regulation. Understanding the integration of metabolism and signaling networks helps reveal how cancer cells metabolically regulate malignant properties and reshape their microenvironment to support tumor growth. This integrative view of metabolism and signaling underscores the complexity and adaptability of cancer cells, highlighting how metabolic reprogramming is actively regulated to confer a growth advantage and enable cancer cells to thrive even in nutrient-poor environments. Given the centrality of metabolism to cancer progression, there is a growing interest in exploring how cancer cells cope with nutrient limitations within the tumor microenvironment. Here, nutrients are often scarce due to poor blood supply, competition among cells or rapid enzymatic consumption. By creating custom-made culture media that precisely control the availability of specific nutrients, researchers can simulate nutrient-poor conditions to investigate which nutrients are critical for cancer cell survival, and understand how cancer cells adapt to nutrient limitations.

The activity of mitochondrial respiration can be assessed through the oxygen consumption rate (OCR) measured, for example, by Seahorse analyzers (Divakaruni et al, 2014). Standard cell culture media contain high levels of buffering agents, such as bicarbonate, which can interfere with the detection of metabolic changes by masking the subtle shifts in pH that Seahorse analyzers measure. To address this, special media are formulated, which are typically bicarbonate-free (Gu et al, 2021). Preparing customized Seahorse media ensures precise adherence to composition requirements, while offering the flexibility to modify specific nutrients and assess their impact on OCR.

Metabolic conditions that alter the availability of nutrients and ATP affect protein biosynthesis, which can be studied through stable isotope labeling by amino acids in cell culture (SILAC) and nascent-proteome experiments by tracking the incorporation of labeled amino acids into newly synthesized proteins. Customized media lacking specific amino acids are essential to ensure exclusive incorporation of labeled amino acids into proteins. Hence, generating one’s own medium lacking the amino acids that will be replaced in the experimental procedure facilitates proteome-wide analysis of changes in protein biosynthesis and provides the flexibility to investigate this across diverse metabolic conditions. Finally, custom-made media enriched with isotopically labeled metabolites enable studying the fates of specific metabolites in cancer cells, as researchers can trace the flow of these molecules through metabolism.

Taken together, custom-made media are helpful for all of these applications in cancer metabolism research. However, purchasing commercially available media that lack specific components is costly due to the specialized manufacturing process, low demand, and bulk ordering requirements. Moreover, it may become entirely unfeasible if companies do not provide full customization options. This can be mitigated by making one’s own medium, which also offers the flexibility to tailor it to the specific needs of the experiment, for example, by omitting single or multiple nutrients or vitamins, or by titrating specific media components. However, preparing cell culture medium from individual components is difficult because compounds have unique solubility, requiring tailored conditions for dissolution. This necessitates the preparation of stock solutions at different concentrations, which is labor-intensive and time-consuming, and must be done under sterile conditions.

**Figure 1.**
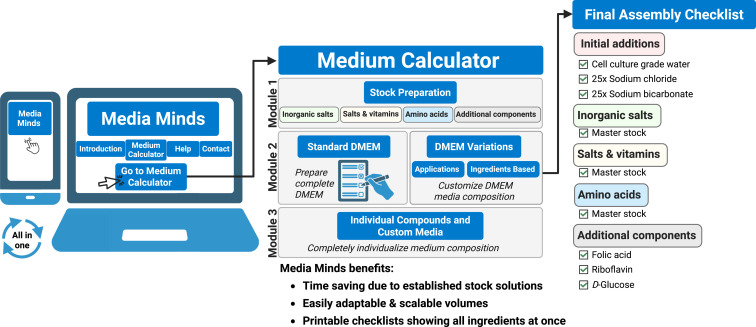
Workflow of “Media Minds” and preparation of self-made cell culture media. The application (desktop and mobile) guides users through three modules: (1) stock preparation: calculate and prepare concentrated stocks of salts, vitamins, amino acids, or additional components; (2) standard DMEM or DMEM variations: assemble complete DMEM or application-specific (e.g., Seahorse, SILAC) or ingredients-based (e.g., amino acid-depleted) variants; (3) individual compounds and custom media: compute weigh-in masses and volumes from molar mass and target concentration for any compound, enabling fully customized media formulations. The tool updates masses, concentrations, and water volumes in real time, and generates printable checklists.

Here, we provide a step-by-step guide to preparing custom-made media, and present “Media Minds,” an online calculator with comprehensive background information (Fig 1). “Media Minds” saves time by automating the calculations, it automatically adjusts volumes based on the amount weighed in for each compound, and it effortlessly adapts to different batch sizes. It can even be used on smart phones in the laboratory to streamline the weighing process. Detailed instructions accompany “Media Minds” and ensure that users not only calculate accurately, but also correctly execute the steps of the protocol, offering detailed guidance for challenging parts of the process.

Finally, we describe an ensemble of metabolic assays performed with custom-made media to provide examples and guidance on how to set up diverse metabolic experiments, which controls to include, how to perform sample preparation, which readouts to measure and how to interpret them.

## Results

### Comparison of glioblastoma cell proliferation in self-made and commercial medium

The preparation of custom-made media addresses a gap in metabolism research where the inability to modify commercial media compositions limits the exploration of nutrient-dependent cellular processes.

Our approach involves creating various media formulations, allowing precise modification or omission of specific components such as critical amino acids (e.g., methionine [Met] or tryptophan [Trp]), vitamins (e.g., nicotinamide [NAM]), and metabolic substrates (e.g., glucose), tailored to experimental needs. This capability is crucial for a range of applications, from assessing ATP production in Seahorse assays, to depleting specific media components for studying nutrient dependency, for proteomics approaches with SILAC labeling, and even for tracing metabolic pathways using heavy-labeled isotopes.

**Figure 2.**
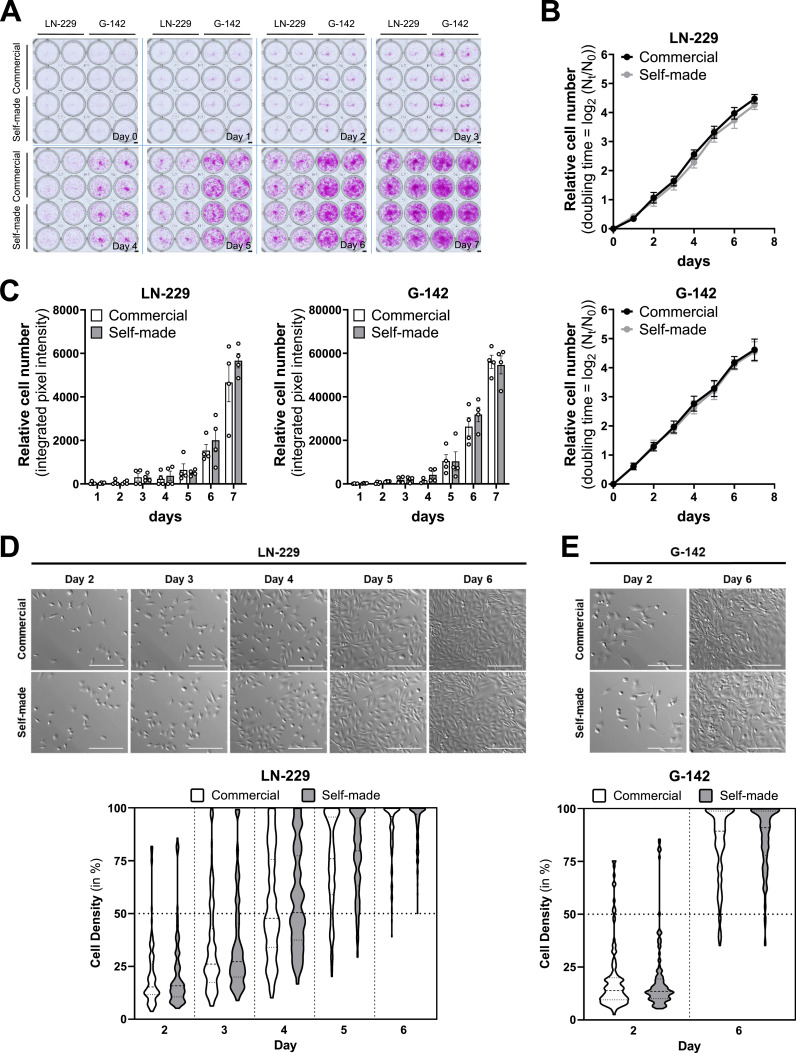
Comparison of commercial and self-made cell culture media shows no differences in cell proliferation. **(A)** Representative images of an SRB assay time course showing LN-229 (left) and G-142 (right) cells cultured in commercial (top) or self-made (bottom) medium over 7 d. n ≥ 5. Scale bar (lower right): 250 μm. **(B)** Relative cell numbers of LN-229 (top) and G-142 (bottom) cultured in commercial (black) or self-made (grey) medium over 7 d, with doubling time calculated from plate-reader measurements at 585 nm and expressed as log_2_ (Nt/N_0_), n ≥ 5. **(C)** Relative cell numbers of LN-229 (left) and G-142 (right) cells cultured in commercial (white) or self-made (grey) medium over 7 d, depicted as integrated pixel intensity, n = 4. **(D)** Representative microscopy images (top) and quantification of cell density (bottom) of LN-229 cells, cultured in commercial (upper row/white) or self-made (lower row/grey) medium for 2–6 d. Images were taken at the same position within the well. Scale bar: 250 μm, n ≥ 3. **(E)** Representative microscopy images (top) and quantification of cell density (bottom) of G-142 cells, cultured in commercial (upper row/white) or self-made (lower row/grey) medium for 2, or 6 d. Images were taken at the same position within the well. Scale bar: 250 μm, n ≥ 3. Data information: **(B, C)** mean ± SEM, two-way ANOVA with a Sidak’s multiple comparisons test. **(D, E)** violin plots, one-way ANOVA with Sidak’s multiple comparisons test. *P* > 0.05, none of the comparisons were significant. Source data are available for this figure.

To validate our self-made medium, we compared the proliferation and morphology of LN-229 and G-142 glioblastoma (GB) cells cultured in commercial or our self-made DMEM. Cell proliferation analysis over 7 d showed no difference in cell density between LN-229 and G-142 cells cultured in commercial or self-made medium. Relative cell numbers assessed via sulforhodamine B (SRB) stainings demonstrated comparable growth kinetics in both cell lines when cultured in either commercial or self-made medium (Fig 2A and B). Microscopy revealed similar relative cell numbers (Fig 2C) as well as morphology of LN-229 and G-142 cells cultured in either media type for up to 6 d (Fig 2D and E). Quantification of cell density from microscopy images confirmed that both the commercial and self-made media equally supported cell growth of both cell lines (Fig 2D and E).

### Assessment of bioenergetic profiles using custom-made media

**Figure 3.**
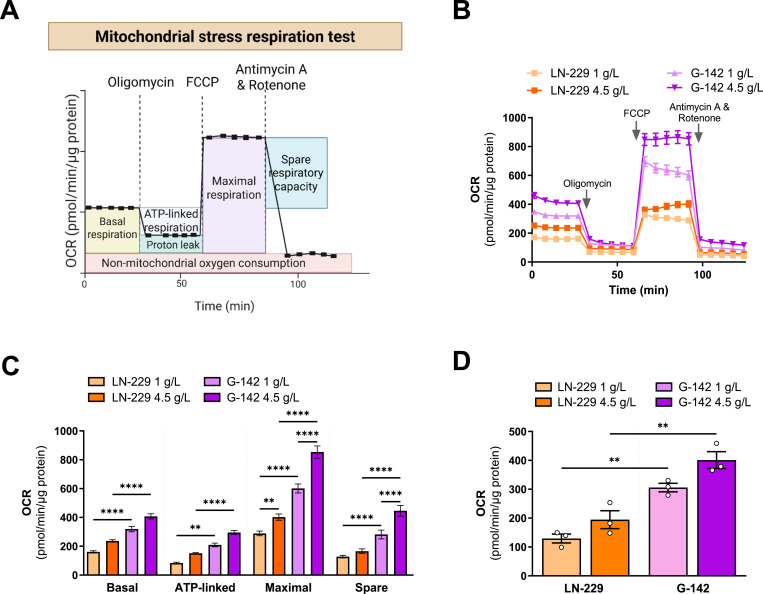
Self-made cell culture medium allows assessment of mitochondrial respiration via Seahorse. **(A)** Schematic overview of the expected profile of a mitochondrial stress respiration test using a Seahorse XF Analyzer. Oligomycin to inhibit ATP synthase, Trifluoromethoxy carbonylcyanide phenylhydrazone (FCCP) to uncouple mitochondrial oxidative phosphorylation, and a combination of Antimycin A and Rotenone to inhibit the mitochondrial electron transport chain. OCR: Oxygen consumption rate. **(B)** OCR profiles of LN-229 cells (squares) and G-142 cells (triangles) after 3 h incubation in self-made DMEM containing either 1 g/liter glucose (light orange for LN-229 cells; light purple for G-142 cells) or 4.5 g/liter glucose (dark orange for LN-229 cells; dark purple for G-142 cells). Arrows indicate sequential injections of metabolic inhibitors to probe cellular responses, n = 3. **(C)** Mitochondrial respiration in LN-229 (glucose 1 g/liter light orange, glucose 4.5 g/liter dark orange) and G-142 (glucose 1 g/liter light purple, glucose 4.5 g/liter dark purple) cells, including basal respiration, ATP-linked respiration, maximal respiratory capacity, and spare respiratory capacity as measured in (B). The bars represent the mean OCR value at the final measurement of each phase, n = 3. **(D)** Average OCR values across the entire measurement time for LN-229 (glucose 1 g/liter light orange, glucose 4.5 g/liter dark orange) and G-142 (glucose 1 g/liter light purple, glucose 4.5 g/liter dark purple) cells, n = 3. Data information: (B, C, D) mean ± SEM, (C, D) One-way ANOVA with Sidak’s multiple comparisons test. **P < 0.01; ****P < 0.0001. Source data are available for this figure.

OCR measurements using the Seahorse XF Analyzer provide a dynamic assessment of mitochondrial respiration (Fig 3A). In the mitochondrial stress test, cells are first monitored under basal conditions, yielding an OCR that reflects the combined oxygen consumption required for ATP synthesis, maintenance of mitochondrial membrane potential, and non-mitochondrial oxygen consumption. Subsequent addition of oligomycin, an inhibitor of ATP synthase (complex V), causes a decrease in OCR corresponding to the fraction of respiration directly coupled to ATP production; this difference between basal OCR and the post oligomycin rate represents ATP-linked respiration. Trifluoromethoxy carbonylcyanide phenylhydrazone (FCCP) is then introduced to collapse the proton gradient across the inner mitochondrial membrane, thereby uncoupling oxidative phosphorylation from ATP synthesis and allowing the electron transport chain to operate at its maximal capacity. Under these conditions, the OCR reaches its highest level, representing maximal respiration. The difference between maximal and basal OCR defines the spare respiratory capacity, representing the ability of mitochondria to respond to increased demand. Finally, the addition of antimycin A (complex III inhibitor) and rotenone (complex I inhibitor) completely shuts down mitochondrial electron transport, revealing the residual OCR due to non-mitochondrial oxygen consumption (Fig 3A).

We used our self-made cell culture medium to measure mitochondrial respiration in LN-229 and G-142 GB cells. To evaluate the influence of glucose availability on OCR, we cultured the cells in media containing either 1 g/liter or 4.5 g/liter glucose. OCR showed distinct respiratory profiles between LN-229 and G-142 cells cultured in media containing either 1 g/liter or 4.5 g/liter glucose (Fig 3B–D). Basal OCR levels were higher in G-142 compared with LN-229 cells, with 4.5 g/liter glucose eliciting higher baseline OCR in both cell lines. As expected, oligomycin caused a marked decline in OCR, indicating inhibition of ATP synthase-dependent respiration, whereas FCCP elicited a pronounced increase in OCR, particularly in G-142 cells at 4.5 g/liter glucose. G-142 cells cultured in 4.5 g/liter glucose exhibited the highest maximal OCR, followed by G-142 at 1 g/liter, with both LN-229 groups displaying lower values. Antimycin A and Rotenone reduced OCR to minimal levels, reflecting non-mitochondrial respiration. Spare respiratory capacity, calculated as the difference between maximal and basal OCR, followed the same pattern as the FCCP response, with greater capacity in G-142 than LN-229 cells and the highest values in G-142 cultured in 4.5 g/liter glucose (Fig 3B and C). Average OCR values mirrored these results, with G-142 exceeding LN-229 under both glucose conditions and a higher glucose concentration leading to a higher average OCR in both cell lines (Fig 3D). These results indicate that modulating glucose availability directly shapes the mitochondrial function of GB cells, with G-142 exhibiting higher oxidative capacity than LN-229. This is consistent with previous findings that GB cells often display both high glycolytic activity and substantial mitochondrial respiration, contributing to tumor aggressiveness and therapy resistance (Sharapova et al, 2024).

### Self-made medium enables analysis of the effects of vitamin B3 depletion on cellular metabolites

**Figure 4.**
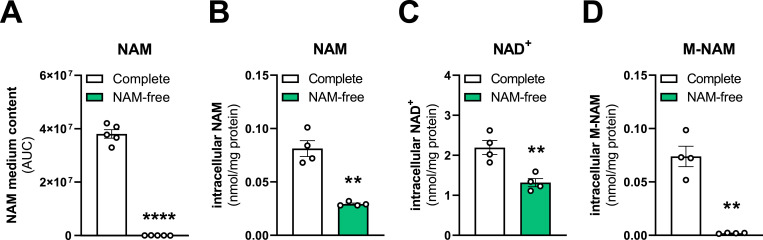
Investigation of the effects of vitamin B3 depletion on cellular metabolites. **(A)** Measurement of nicotinamide (NAM) content in self-made DMEM containing 33 μM NAM (complete, white) or lacking NAM (NAM-free, green), quantified by semi-targeted metabolomics. NAM is quantified as area under the curve (AUC) in arbitrary units. n = 5. **(B)** Measurement of intracellular NAM in LN-229 cells cultured in self-made complete (white; 33 μM NAM) or NAM-free (green) culture media for 24 h, measured by LC-MS/MS. n = 4. **(C)** Measurement of nicotinamide adenine dinucleotide (NAD^+^) as described in (B). n = 4. **(D)** Measurement of 1-methylnicotinamide (M-NAM) as described in (B). n = 4. Data information: (A, B, C, D) mean ± SEM, paired *t* tests. **P < 0.01, ****P < 0.0001. Source data are available for this figure.

Cell culture media commonly contain NAM, the amide form of vitamin B3, but usually lack other vitamin B3 forms such as nicotinic acid or nicotinamide riboside (Eagle, 1955; Eagle & Habel, 1956; Dulbecco & Freeman, 1959). Using our custom-made medium, we investigated how removing NAM from the medium affects metabolite levels in LN-229 cells (Fig 4A–D). First, we confirmed that our NAM-free medium truly lacked NAM by measuring its levels in the medium (Fig 4A). We next measured intracellular NAM levels in LN-229 cells grown for 24 h under either NAM-proficient or NAM-deficient conditions. Cells cultured in NAM-free medium showed a 64% drop in intracellular NAM levels, confirming effective depletion (Fig 4B). This depletion directly impacted metabolic pathways, in which NAM serves as a precursor. NAM is converted to nicotinamide adenine dinucleotide (NAD^+^) in a two-step process through the enzyme nicotinamide phosphoribosyltransferase (NAMPT), which transforms NAM into nicotinamide mononucleotide (NMN), and the nicotinamide mononucleotide adenylyltransferases (NMNATs), which then convert NMN to NAD^+^. Our measurements revealed a 40% decrease in intracellular NAD^+^ following NAM removal from the medium (Fig 4C). We also analyzed the levels of 1-methylnicotinamide (M-NAM), the methylated form of NAM generated by the enzyme nicotinamide N-methyltransferase (NNMT). In NAM-free conditions, intracellular M-NAM levels dropped to nearly undetectable levels (Fig 4D), likely due to insufficient NAM availability as a substrate for NNMT.

Our results demonstrate that removing NAM from the medium directly translates into decreased intracellular NAM, reduced NAD^+^ and depletion of M-NAM. The observation that NAD^+^ is reduced while M-NAM is depleted, aligns with reports indicating that NAMPT exhibits a much lower Km (<1 μM) than NNMT (∼430 μM) for NAM (Kang-Lee et al, 1983; Aksoy et al, 1994; Revollo et al, 2007; Burgos & Schramm, 2008; Liu et al, 2018). These data indicate that under NAM scarcity, the high-affinity NAMPT enzyme remains active, while the lower-affinity NNMT enzyme becomes inactive due to lack of sufficient substrate (Narayanan et al, 2025). Overall, this use case demonstrates how making one’s own medium, in which specific components can be omitted, enables studying the impact of vitamin depletion on key metabolic pathways. By selectively omitting NAM, we could investigate how different enzymes compete for the same substrate and examine effects on downstream metabolites. This enables dissecting nutrient-driven regulation of cellular metabolism and offers a platform for experiments involving targeted depletion of vitamins and cofactors with broad implications for metabolism and epigenetics.

### Self-made medium enables investigation of protein methylation upon methionine limitation

**Figure 5.**
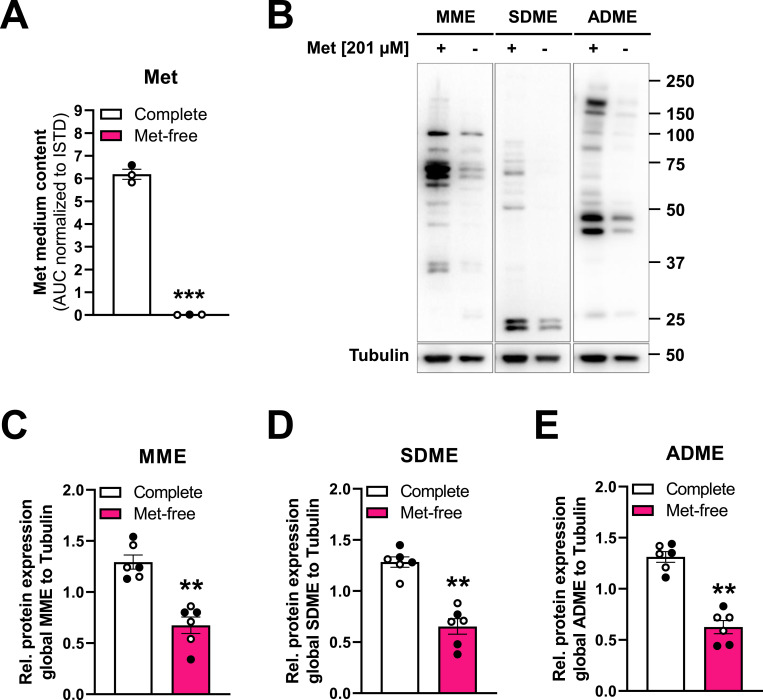
Investigation of protein methylation upon methionine limitation. **(A)** Methionine (Met) content in self-made (white dots) or commercial (black dot) DMEM containing 201 μM Met (complete, white) or lacking Met (Met-free, pink), measured by targeted metabolomics. Met quantified as area under the curve (AUC) in arbitrary units, normalized to a heavy-labeled Met-^13^C,^15^N internal standard. n = 3. **(B)** Representative immunoblot of global mono-methylated (MME), symmetrically di-methylated (SDME), or asymmetrically di-methylated (ADME) protein arginine residues in LN-229 cells cultured in complete or Met-free medium for 48 h. Representative of n = 6. **(C)** Quantification of global MME signal intensities (relative to tubulin) in complete (white) or Met-free (pink) media as in (B), n = 6; commercial (black dots, n = 3) and self-made (white dots, n = 3) DMEM shown. **(D)** Quantification of global SDME signal intensities as described in (C), n = 6. **(E)** Quantification of global ADME signal intensities as described in (C), n = 6. Data information: (A, C, D, E): mean ± SEM, paired *t* test, **P < 0.01, ***P < 0.001. Source data are available for this figure.

Self-made culture media also allow investigating the effects of amino acid starvation. Here, we demonstrate a possible application of Met-free medium using GB cells as a model system (Fig 5A–E). Commercially available DMEM typically contains 201 μM Met and cystine, but no cysteine or homocysteine (Eagle, 1955; Eagle & Habel, 1956; Dulbecco & Freeman, 1959). To determine the Met-mediated effects, we prepared self-made media lacking Met. First, we confirmed that the Met-free medium we prepared lacked Met using a targeted liquid chromatography-mass spectrometry (LC-MS/MS) approach (Fig 5A). The essential amino acid Met is a precursor for S-adenosylmethionine (SAM), which serves as a methyl donor for all methyltransferases, including those for protein methylation (Guccione & Richard, 2019; Wu et al, 2021). To investigate the effect of Met limitation on protein methylation, we performed immunoblot analysis using antibodies against mono- and di-methylated protein arginine residues (Fig 5B). Met-deprived cells showed a significant reduction in mono-methylation (MME, Fig 5C), symmetrical- (SDME, Fig 5D) and asymmetrical-di-methylation (ADME, Fig 5E) of protein arginine residues by ∼ 52%, 50%, or 48%, respectively. Both self-made and commercially available amino acid-free medium to which all amino acids except Met were supplemented, exhibited similar reductions in the respective methylation patterns (Fig 5C–E), further confirming the reliability and reproducibility of our self-made medium. The observed reduction in protein arginine methylation suggests that Met depletion limits the function of protein arginine methyltransferases (PRMTs). However, it must be taken into account that because the medium lacks cysteine and homocysteine, the effect of Met deprivation may be more pronounced than in physiological conditions. These results highlight the broad range of possible applications for self-made media in metabolic, epigenetic, and cancer research in particular with regard to studying the function and importance of single amino acids in these processes.

### Self-made medium enables investigation of the impact of tryptophan limitation on the integrated stress response

**Figure 6.**
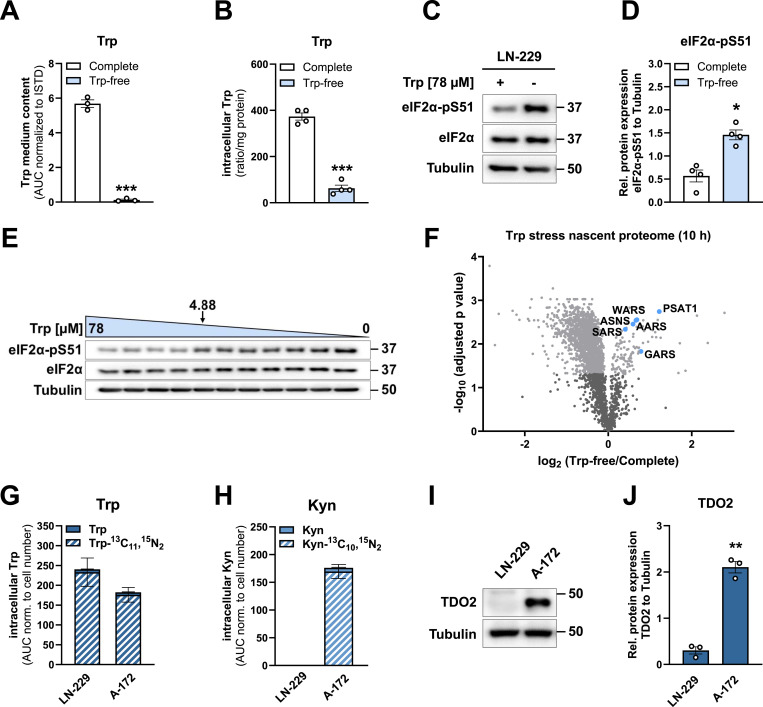
Application of self-made media containing defined tryptophan levels or heavy-labeled tryptophan. **(A)** Tryptophan (Trp) content in self-made DMEM containing 78 μM Trp (complete, white) or lacking Trp (Trp-free, light blue), measured by LC-MS/MS. Trp quantified as area under the curve (AUC) in arbitrary units, normalized to heavy-labeled Trp-^13^C_11_,^15^N_2_ internal standard, n = 3. **(B)** Intracellular Trp content in LN-229 cells cultured in self-made complete (white) or Trp-free (light blue) culture media for 24 h, measured by LC-MS/MS. n = 4. **(C)** Representative immunoblot of eIF2α-pS51 and total eIF2α in LN-229 cells, cultured in complete or Trp-free medium for 48 h. Representative of n = 4. **(D)** Quantification of eIF2α-pS51 protein signal intensities (rel. to Tubulin) in LN-229 cells, cultured in complete (white) or Trp-free (light blue) media for 48 h, n = 4. **(E)** Representative immunoblot of eIF2α-pS51 and total eIF2α in LN-229 cells cultured with decreasing Trp concentrations (78–0 μM) for 48 h. Representative of n = 5. **(F)** Analysis of the nascent proteome in Trp-depleted LN-229 cells. Volcano plot showing Trp depletion-dependent changes in the newly synthesized proteome. Significantly up- (124) or down-regulated (1,800) proteins with an adjusted *P*-value < 0.05 are shown in light grey (−log_10_ *P*-value > 1.301). Key up-regulated proteins in response to Trp stress are colored in blue: phosphoserine aminotransferase (PSAT1), asparagine synthetase (ASNS), tryptophanyl-tRNA ligase (WARS), alanine-tRNA ligase (AARS), serine-tRNA ligase (SARS), glycyl-tRNA ligase (GARS). **(G)** Measurement of intracellular non-labeled (Trp) and heavy-labeled (Trp-^13^C_11_,^15^N_2_, dashed) Trp in LN-229 (n = 3) and A-172 (n = 2) cells, cultured in self-made DMEM for 72 h, measured by LC-MS/MS. Shown are the areas under the curve (AUC) in arbitrary units of Trp normalized to cell number. **(H)** Measurement of kynurenine (Kyn) and heavy-labeled (Kyn-^13^C_10_,^15^N_2_, dashed) Kyn in LN-229 (n = 3) and A-172 (n = 2) cells, cultured in self-made DMEM for 72 h, measured by LC-MS/MS. Shown are the areas under the curve (AUC) in arbitrary units of Kyn normalized to cell number. **(I)** Representative immunoblot of TDO2 in LN-229 and A-172 cells, cultured in complete self-made DMEM. Representative of n = 3. **(J)** Quantification of TDO2 signal intensities (rel. to Tubulin) in LN-229 and A-172 cells, cultured in complete self-made DMEM (n = 3). Data information: (A, B, D, G, H, J): mean ± SEM, (A, B, D, J): paired *t* test, *P < 0.05, **P < 0.01, ***P < 0.001. Source data are available for this figure.

To investigate the effect of other essential amino acids on metabolic processes, we also prepared self-made media containing or lacking Trp (Fig 6A). Trp is the least abundant essential amino acid and DMEM contains 78 μM Trp, which reflects human plasma levels (Eagle, 1955; Eagle & Habel, 1956; Dulbecco & Freeman, 1959; Psychogios et al, 2011). In our self-made media, we determined Trp levels using targeted LC-MS/MS. Indeed, we confirmed the absence of Trp in the Trp-free medium (Fig 6A). In GB cells cultured in Trp-free medium for 24 h, intracellular Trp levels were reduced by ∼80%, compared with cells cultured in Trp-containing medium (Fig 6B).

Trp is an essential amino acid and is therefore vital for protein synthesis. Amino acid shortage is well-known to block protein translation, acting as a protective mechanism to shield cells from nutritional stress, and conserve building blocks and energy (Richard et al, 2009). This pro-survival response is typically regulated by the integrated stress response (ISR), a signaling pathway involving four stress kinases that phosphorylate the eukaryotic initiation factor 2 subunit alpha eIF2α at serine 51 (eIF2α-pS51) in response to various stress stimuli (Ernst et al, 1987; Hershey, 1989; Clemens, 1994). Indeed, phosphorylation of eIF2α was induced in GB cells that were cultured in Trp-free medium for 48 h (Fig 6C and D). Furthermore, our self-made medium provides the opportunity to titrate extracellular Trp concentrations, enabling precise determination of the dynamics and the threshold for ISR activation. In GB cells cultured with extracellular Trp concentrations ranging from 78 to 0 μM, phosphorylation of eIF2α-pS51 was induced as soon as extracellular Trp fell below 4.88 μM at 48 h (Fig 6E), a finding which is in line with data upon 24 h of Trp depletion previously published by our group (Holfelder et al, 2024 *Preprint*).

### Self-made media for nascent-proteome analysis

To further investigate the impact of Trp stress on protein biosynthesis, we analyzed the newly translated proteins upon Trp deprivation using a SILAC-based nascent-proteome approach (Eichelbaum et al, 2012; Borteçen et al, 2023) (Fig 6F). This method requires a Met-, Arg-, and Lysine (Lys)-free medium: Met is replaced by the clickable Met analog 4-Azido-*L*-homoalanine (AHA) to enrich newly synthesized proteins, and Arg and Lys are replaced by their heavy- and intermediate-labeled counterparts to distinguish between the conditions of interest. To assess the effects of Trp stress on protein biosynthesis, we therefore created a medium lacking Met, Arg, Lys and in addition Trp to enable subsequent nascent-proteome analysis.

Cells were cultured for 10 h in self-made media either containing or lacking Trp. During the final 6 h, pulse labeling was conducted using self-made media supplemented with AHA and either heavy- or intermediate-labeled Arg and Lys to assess newly synthesized proteins and to differentiate between control and Trp-free conditions. Trp stress up-regulated 124 proteins (Fig 6F). The top up-regulated proteins in response to Trp depletion are involved in the stress response: We observed the induction of a specific set of tRNA aminoacyl synthetases, namely tryptophanyl-tRNA ligase (WARS), alanine-tRNA ligase (AARS), serine-tRNA ligase (SARS), and glycyl-tRNA ligase (GARS). Furthermore, Trp depletion induced ISR target proteins including phosphoserine aminotransferase (PSAT1) and asparagine synthetase (ASNS). In summary, creating customized culture media with precise control over the composition of natural, modified, and labeled amino acids represents a powerful tool for advanced proteomics approaches, including metabolic labeling and nascent-proteome analysis.

### Self-made medium enables tracing of metabolites to reveal distinct activity of metabolic pathways

In addition to studying protein synthesis, Trp-free medium facilitates metabolite tracing experiments. The substitution of Trp with its heavy isotope-labeled counterpart enables precise tracking of downstream metabolic fluxes (Fig 6G and H). Heavy-labeled Trp (Trp-^13^C_11_,^15^N_2_) was readily taken up by LN-229 and A-172 GB cells (Fig 6G). Interestingly, the conversion of heavy-labeled Trp to kynurenine (Kyn) occurred only in A-172 but not in LN-229 cells (Fig 6H). In GB cells, Kyn is mainly generated through the Trp-catabolizing enzyme tryptophan-2,3-dioxygenase (TDO2) (Opitz et al, 2011). Consistent with the difference in Kyn levels, the enzyme responsible for Kyn generation, TDO2, was expressed in A-172 cells but hardly in LN-229 cells (Fig 6I and J). Taken together, self-made media facilitate studying metabolic pathways in detail by stable-isotope tracing.

## Discussion

The preparation of custom-made cell culture media enables precise modulation of medium composition, thereby facilitating the investigation of diverse metabolic processes. Another major advantage of preparing medium from scratch is its cost-effectiveness. Self-prepared medium is ∼15 times less expensive than commercially available formulations. This estimation is based on the individual ingredient costs of Trp-free DMEM: the total cost of the components amounts to roughly €5.50 per 500 ml bottle, whereas commercially available Trp-free medium costs ∼€85 per 500 ml (based on a bulk order from Gibco, Thermo Fisher Scientific, in 2025). However, when considering additional steps such as sterile filtration and basic quality-control tests (e.g., pH, osmolarity, and endotoxin levels), the cost ratio changes, with Trp-free self-prepared medium being ∼4 times less expensive than the commercial equivalent.

Despite these advantages, preparing cell culture medium in-house also poses certain challenges. Preparing the necessary stock solutions alone can take 2–3 d, ideally done by two people to share the workload. Solubility differences between components demand multiple stock concentrations to ensure proper dissolution. Without our guidance, users would need to test and optimize these concentrations themselves—a time-consuming process—but we here provide fully tested stock solution formulations. Mixing the correct stocks for different media applications can take up to 1.5 h, and requires attention to sterility and pH. This process is also prone to error—a mistake involving a single ingredient can compromise an entire batch. In addition, manual calculations can lead to errors, especially when converting between units or scaling recipes. While general molarity calculators exist, they are not tailored to the complexity of medium preparation. Our “Media Minds” calculator not only supports accurate calculations specific for media preparation, but also guides users through the entire process and includes a checklist to ensure that no component is accidentally left out, thereby saving time and reducing errors (Fig 1).

In this study, we used DMEM as a model system to demonstrate the feasibility of preparing customized cell culture media for cancer metabolism research. While it is well recognized that DMEM is a nutrient-rich medium designed to support robust cell growth, it does not capture the complexity of physiological conditions (Psychogios et al, 2011). However, it remains one of the most widely used media in biomedical research due to its defined composition, ease of modification, and historical precedence in cell culture studies (Ackermann & Tardito, 2019; Cantor, 2019; Wijerathna-Yapa et al, 2025). Importantly, our approach was not intended to advocate for DMEM as the optimal medium for studying cancer metabolism but rather to provide a framework for media customization that can be extended to other formulations. Given the increasing emphasis on metabolically accurate cell culture conditions, researchers can adapt our protocol to generate media formulations that more closely mimic in vivo nutrient environments, such as Plasma-like Medium (PLM), Human Plasma-like Medium (HPLM), or tumor-microenvironment-adapted formulations such as Tumor Interstitial Fluid Medium (TIFM) (Cantor et al, 2017; Vande Voorde et al, 2019; Golikov et al, 2021; Golikov et al, 2022; Apiz Saab et al, 2023; El Shami et al, 2023 *Preprint*). Through preparation of self-made medium, researchers can mimic the tumor microenvironment more accurately, adjust specific nutrient levels, and investigate how cancer cells respond to metabolic stressors (Sullivan et al, 2019; Abbott et al, 2024; Sivanand et al, 2024)—an increasingly important topic in cancer metabolism research.

However, media preparation alone is not enough, careful design, and execution of experiments is essential to extract meaningful insights into cancer metabolism. As a quick reference, we have therefore summarized “key considerations for cancer metabolism experiments” in Box 1, and we will further elaborate on these points and discuss them in the following section.

Box 1 Key considerations for cancer metabolism experiments.

***Metabolically characterize the conditions you are working in:***

• Is the substrate for the enzyme you wish to study present (contained in your media or produced by the cells)?
• Confirm whether the media composition is altered the way you intend.
• Validate enzyme inhibition by measuring the products (and substrates) of the enzymatic reaction you wish to block.
• Use the correct vehicle controls (e.g., solvents can have major metabolic effects).

***Metabolite measurements:***

• Pre-test interventions to exclude major effects on cell viability that may mask the metabolic effects you wish to study.
• Perform independent biological replicates.
• Ensure metabolite extraction is adapted to unstable metabolites.
• Measure intra- and extracellular metabolite concentrations.
• Use heavy-labeled standards and check retention time and MS spectra for identification of compounds.
• Normalize metabolic data to cell number or total protein to avoid artifacts due to cell proliferation differences.

To accurately interpret metabolic alterations, it is crucial to first establish whether the medium and cellular environment support the metabolic pathways under investigation. One of the first considerations is to determine whether the substrate of the enzyme being studied is present in the system, either supplied in the culture medium or produced endogenously by the cells. If its substrate is unintentionally absent, the enzyme’s metabolic role may be underestimated, potentially leading to inaccurate conclusions.

When creating metabolite-depleted media, it is crucial to ensure that the omitted metabolite is not unintentionally reintroduced through serum, supplements, or metabolic interconversion by the cells. Strategies such as using dialyzed FBS (store-bought or self-made), analyzing supplement compositions, eliminating structurally similar metabolites, and performing metabolomic validation ensure that the culture conditions accurately reflect the intended nutrient restriction. Without these confirmation steps, experiments may yield misleading results, as cells could still access the omitted nutrient, compromising the expected metabolic effects.

Another critical factor in experimental design is validating enzyme inhibition. If an experiment involves pharmacologically blocking an enzyme to study its metabolic impact, inhibition must be confirmed by measuring the products and, if possible, also the substrates of the enzymatic reaction. A successful inhibition results in product depletion and often substrate accumulation, serving as a direct biochemical confirmation of the enzyme’s activity being disrupted. When using pharmacological inhibitors, it is critical to validate functional inhibition rather than relying solely on mRNA or protein expression data. Measuring substrate and product levels, enzymatic activity, or metabolic flux provides a more accurate assessment of enzyme function. Moreover, since inhibitor efficacy can vary between cell types due to differences in permeability, expression levels, and compensatory mechanisms, optimal inhibitor concentrations must be empirically determined. Without these considerations, there is a risk of misinterpreting both the presence and absence of metabolic changes either attributing effects incorrectly to enzyme inhibition or concluding that the enzyme is not functionally relevant when, in reality, it was not effectively inhibited.

In addition, the use of appropriate vehicle controls is necessary to rule out unintended metabolic effects from solvent-based inhibitors. Many enzyme inhibitors are dissolved in DMSO, ethanol, or methanol, which—even at low concentrations—can significantly impact metabolism, particularly lipid metabolism, mitochondrial function, and redox balance (Ma et al, 2018). Including appropriate solvent controls ensures that the observed metabolic effects are due to enzyme inhibition rather than solvent-induced artifacts.

Once the experimental conditions are properly characterized, accurate metabolite measurements are required to extract meaningful data. One important step is pretesting experimental interventions to ensure that metabolic changes are not simply secondary effects of cell death or compromised viability. If a treatment significantly reduces cell viability, metabolite levels may shift due to nonspecific cell stress responses, apoptosis, or necrosis rather than the intended metabolic alteration.

To generate reproducible and biologically relevant data, it is essential to perform independent biological replicates rather than relying solely on technical replicates. Biological replicates—samples derived from independent cell cultures—help account for passage number effects, and subtle changes in experimental conditions or culture conditions that could influence metabolism.

Another major consideration is the stability of metabolites during extraction. Many metabolites, particularly those in glycolysis, the tricarboxylic acid cycle, and nucleotide metabolism (Scoazec et al, 2014; Dienel, 2020; Rost et al, 2020), are highly labile and degrade rapidly once cells are lysed. Proper quenching protocols, rapid sample processing, and adequate storage conditions are essential to preserve metabolite stability and avoid artificial alterations in metabolic profiles.

Both intracellular and extracellular metabolite concentrations should be measured to differentiate between changes in metabolite transport and alterations in metabolite generation or degradation. If only intracellular metabolite levels are assessed, a decrease in a specific metabolite could be mistakenly interpreted as increased consumption or degradation, when in reality, it might be due to enhanced export out of the cell. Conversely, an increase could result from reduced export rather than increased synthesis. By simultaneously quantifying intra- and extracellular metabolites, researchers can determine whether metabolic changes arise from altered cellular uptake, secretion, or intracellular processing. For instance, if an inhibitor is expected to block an enzyme in a metabolic pathway, but extracellular metabolite concentrations change while intracellular levels remain constant, it may suggest disrupted transport rather than enzymatic inhibition. Different cell types may exhibit varying transporter expression, further emphasizing the need for a comprehensive analysis to avoid misinterpretation of metabolic alterations.

To ensure accurate metabolite identification and quantification, the use of heavy-labeled internal standards (ISTDs) is recommended. By incorporating stable isotope-labeled metabolites early in the sample preparation process, researchers can account for metabolite loss during extraction, processing, and handling, ensuring accurate recovery. Because of these ISTDs undergo the same preparation steps as endogenous metabolites, they help normalize extraction efficiency and sample recovery, minimizing artifacts introduced by incomplete metabolite retention or degradation. During mass spectrometry-based metabolomics, they further correct for instrumental variability, matrix effects, and signal fluctuations, enhancing the precision and reproducibility of metabolite quantification. This approach ensures that observed metabolic differences reflect true biological variations rather than technical inconsistencies. Checking retention times and mass spectra for each compound ensures that metabolite identification is accurate and not confounded by overlapping peaks or unexpected ion fragmentation patterns. To explore this topic in greater detail, Johannes Meiser and Christian Frezza have recently addressed common challenges in metabolomics data interpretation and offer guidelines for accurate data presentation (Meiser & Frezza, 2024).

Finally, metabolic data should be normalized to cell number or total protein content to avoid misleading conclusions due to variations in cell proliferation. Since metabolic activity is tightly linked to cell density, failing to normalize metabolite levels could result in artifacts where observed changes are due to differences in cell number rather than true metabolic reprogramming.

In summary, customizing cell culture media provides a valuable tool for studying cancer metabolism under controlled conditions, but careful metabolic characterization is essential to ensure that experimental findings are meaningful. Controlling enzyme activity, confirming nutrient availability, and ensuring the intended media composition are critical steps before conducting downstream analyses. Once these conditions are validated, metabolite measurement protocols, including viability controls, stability assessments, and normalization strategies, ensure that metabolic alterations are accurately quantified and interpreted. By implementing these best practices, researchers can minimize the risk of technical artifacts influencing experimental conclusions and generate reproducible, biologically relevant insights into cancer metabolism.

## Materials and Methods

To reproduce the cell culture medium formulations and experimental procedures provided in this study, we present all the reagents and tools with detailed ordering information in Table S1.

Table S1. Structured methods: reagents and tools table.

### Cell cultivation and preparation for experimental procedures

LN-229 and A-172 GB cells were purchased from ATCC. NCE-G142 (G-142) GB cells were a kind gift from Dr. Katrin Lamszus (University Hospital Hamburg-Eppendorf). All cells were maintained in commercial, 4.5 g/liter glucose DMEM (Gibco, Thermo Fisher Scientific) supplemented with 10% FBS (Thermo Fisher Scientific), 1 mM sodium pyruvate (Gibco, Thermo Fisher Scientific) and 2 mM *L*-glutamine (Gibco, Thermo Fisher Scientific) and optionally with 100 U/ml penicillin and 100 μg/ml streptomycin (Gibco, Thermo Fisher Scientific). Cells were cultured at 37°C in a humidified incubator (SANYO Electric Co., Ltd.) with 5% CO_2_. Mycoplasma testing was routinely performed using a mycoplasma testing kit from Minerva Biolabs GmbH. When cells reached 90–100% confluency, they were washed once with phosphate buffered saline (PBS, Gibco, Thermo Fisher Scientific) and then detached using trypsin-ethylendiaminetetraacetic acid (EDTA, Gibco, Thermo Fisher Scientific). After detachment, cells were resuspended in fresh complete medium and transferred to new culture dishes.

For experiments, cells were centrifuged at 300*g* for 5 min, counted, and seeded at the required densities in fresh, complete medium. Cells were used between passage number 5 and 20 to minimize variations.

### Preparation of custom-made cell culture medium

Here, we introduce a step-by-step protocol to prepare custom-made DMEM (Table S2). Furthermore, we provide the cell culture media calculator “Media Minds,” an interactive online tool that assists in not only preparing custom-made DMEM but that can also be used to generate other cell culture media formulations.

Table S2. Preparation of custom-made DMEM variations.

To prepare custom-made cell culture media, we recommend the following steps:

(1) Preparation of individual stocks for every compound tailored to its solubility and the appropriate solvent.
(2) Preparation of master stocks of similar compounds that can be dissolved and stored together (using the individual stocks from (1)).
(3) Assembly of the respective individual or master stocks for the desired medium and addition of cell culture-grade water.
(4) pH adjustment and sterile filtration.
(5) Supplementation with instable compounds such as FBS, *L*-glutamine, sodium pyruvate, and antibiotics (penicillin-streptomycin) immediately before medium use.

#### Preparation of individual stocks for custom-made medium

To save time while preparing the medium for individual experiments and to remain adaptable to individual research questions, individual stocks of all cell culture medium components were prepared (Table S2).

The procedure of weighing in the pure substances was carried out as follows: First, the weight m_1_ of the empty conical tube was determined. Second, the conical tube was opened under a sterile fume hood, the respective powder was transferred, the tube was closed, and weighed once more to determine m_2_. The actual weight of the substance inside the conical tube is calculated as m_2_—m_1_. This value can be directly transferred to our cell culture media calculator “Media Minds,” which automatically determines the volume that is required to prepare the stock solutions. Note: Use sterile, cell culture-grade solvents for preparing stock solutions and pay attention to the specific storage requirements (e.g., light sensitivity, temperature constraints). Documenting lot numbers, preparation dates, and any pH adjustments for each stock solution are strongly recommended to trace potential inconsistencies in subsequent experiments.

All amino acids, vitamins, carbohydrates, inorganic salts, and buffering agents required for DMEM generation as well as information about recommended stock concentrations, solvents, and storage temperatures are listed in Table S2.

Individual components shown in Table S2 completely dissolve in the given solvent at the recommended stock concentration and were designed to take up the minimal possible volume to save space. However, if necessary, components can always be dissolved at lower concentrations. When preparing alternative cell culture media formulations, it is essential to test the solubility of each component in advance. Note: After thawing, solutions may precipitate but can easily be redissolved by vortexing or gentle heating (e.g., using a water bath at 37°C). Be sure to check for any color changes (especially with riboflavin [Sigma-Aldrich], as it can degrade or oxidize if exposed to direct light or if repeatedly thawed). In addition, stocks of light-sensitive compounds need to be protected from light exposure by wrapping tubes in aluminium foil or storing them in a dark container immediately after preparation.

Components can either be stored as individual stocks or can be combined as “master stocks,” depending on the media formulation.

#### Preparation of master stocks for custom-made medium

To simplify cell culture media preparation, individual compounds can be combined and stored as master stocks, depending on their solubility. Here, we explain the preparation of three master stocks: 10x amino acid master stock, 100x salts and vitamins master stock and 100x inorganic salts master stock. If one uses complete medium without leaving out a single component, then all three master stocks can be used. However, if one alters the media composition, for example, by titrating or leaving out a certain amino acid, then only the salts and vitamins and the inorganic salts master stock can be used, but not the amino acids master stock. In that case, all other amino acids need to be added as individual components using their respective individual stock. Note: Not all media components can be combined in one of the three master stocks. Those that have to be added as individual compounds are labeled as “Additional Components” in Table S2 (marked in grey).

##### Preparation of 10x amino acid master stock

All amino acids can be stored in a 10x amino acid master stock. Therefore, all amino acids were first dissolved according to their individual solubility as described in Table S2. Well-soluble amino acids such as glycine (Sigma-Aldrich), *L*-arginine (Arg) base (Genaxxon), *L*-histidine hydrochlorid monohydrate (Genaxxon), *L*-lysine (Lys) monohydrochloride (Sigma-Aldrich), *L*-methionine (Met, US biological) were dissolved at 1,000x individual stocks (light blue) in cell culture-grade water (Corning). Due to the poor solubility in water, *L*-cystine dihydrochloride (Sigma-Aldrich) was dissolved as 1,000x individual stock in 1 M HCl (light blue). The other amino acids were prepared as individual 100x stocks (marked in blue): *L*-isoleucine (Genaxxon), *L*-leucine (Sigma-Aldrich), *L*-phenylalanine (Carl Roth), *L*-serine (Genaxxon), *L*-threonine (Sigma-Aldrich), *L*-tryptophan (Trp, Sigma-Aldrich), *L*-tyrosine disodium salt dihydrate (Thermo Fisher Scientific), *L*-valine (Genaxxon). 1,000x and 100x individual stocks were diluted 1:100 or 1:10 to obtain the 10x amino acid master stock (dark blue). *L*-glutamine (Gibco, Thermo Fisher Scientific) was stored separately due to its instability and was freshly supplemented to the medium immediately before experiments. The 10x amino acid master stock was stored in 50 ml sized aliquots at −20°C. Note: Make sure to dissolve as many of the amino acids in water instead of acidic or basic solutions to prevent pH shifts and acid-base reactions. The 100x Lys (Sigma-Aldrich) stock requires thorough mixing to fully dissolve in water.

##### Preparation of 100x salts and vitamins master stock

Choline chloride, *D*-calcium pantothenate, nicotinamide (NAM), pyridoxine hydrochloride, thiamine hydrochloride, and *i*-inositol (all from Sigma-Aldrich) were dissolved as individual 1,000x stocks in cell culture-grade water (Corning) (Table S2, marked in yellow). Due to solubility reasons, folic acid and riboflavin were kept as 100x individual stocks dissolved in 1 M sodium hydroxide (NaOH) and cell culture-grade water, respectively (Table S2, marked in grey). Note: Folic acid and riboflavin both appear yellow when dissolved in the given solvents.

The salts were dissolved in cell culture-grade water (Corning) as follows: 2,000x iron(III) nitrate nonahydrate (Sigma-Aldrich), 1,000x calcium chloride dihydrate and 1,000x potassium chloride (both from Sigma-Aldrich) (Table S2, marked in yellow). Note: Potassium chloride requires thorough stirring and gentle heating at 50°C for 20 min to completely dissolve. Crystallization may occur upon cooling and can be reversed by stirring and reheating the solution.

To prepare the 100x salts and vitamins master stock, all 1,000x and 2,000x individual stocks of the salts and vitamins were combined and hereby diluted 1:10 or 1:20 to obtain a yellow colored 100x master stock. The 100x salts and vitamins master stock was subsequently stored in aliquots of 5 ml at −20°C.

##### Preparation of 100x inorganic salts master stock

The 100x inorganic salts master stock only contains two salts, magnesium sulfate (AppliChem) and sodium dihydrogen phosphate dihydrate (Merck Millipore) (Table S2, marked in green) that may oversaturate the 100x salts and vitamins master stock. Both compounds were first dissolved as 200x individual stocks in cell culture-grade water (Corning) and then combined and diluted 1:2 to obtain the 100x inorganic salts master stock, which was stored in aliquots of 5 ml at −20°C. In case of precipitation, the 100x inorganic salts master stock was gently warmed and thoroughly mixed before use. Note: Due to their high salt concentration, sodium bicarbonate and sodium chloride (both from Sigma-Aldrich) were prepared and kept as individual 25x stocks and were stored at RT (Table S2, marked in grey).

#### pH adjustment and final supplementation of custom-made medium

**Figure S1.**
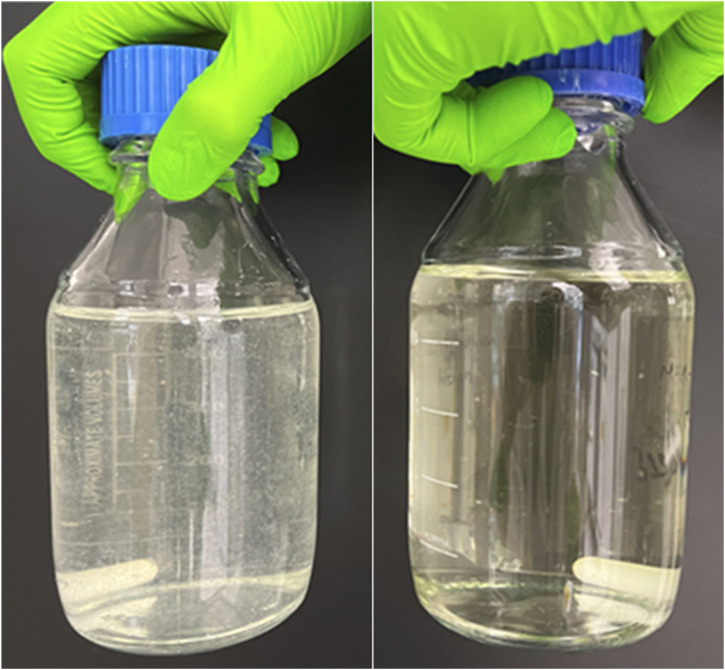
Two bottles of the same custom-made DMEM with pH 9 (left) or pH 8.1 (right).

For the final assembly of all media components, first the required volume of cell culture-grade water (Corning) needs to be determined. For 500 ml custom-made complete DMEM 385 ml of water are needed (Table S2, tick off list). Note: Also, our Cell Culture Media Calculator “Media Minds” is designed to calculate the required volume of cell culture-grade water when using the respective tick off list. Only then the required amount of all three master stocks and the amounts of the additional components ([1] that were not included in one of the master stocks, [2] that are stable and should be added before filtration) (folic acid, riboflavin, sodium bicarbonate, sodium chloride, and *D*-glucose [Sigma-Aldrich]), are added. The medium then has a slightly basic pH and might appear cloudy (Fig S1, left bottle). However, the medium becomes clear as soon as the pH is adjusted to 8.1–8.2 using hydrochloric acid (HCl, Carl Roth) (Fig S1, right bottle). After pH adjustment, the medium is sterile-filtered and stored at 4°C. Note: When cell culture media is stored in a sealed bottle at RT and exposed to atmospheric air (≈0.04% CO_2_), its pH is typically around 8.0. It only drops to physiological pH (∼7.2–7.4) once it is placed in a 5% CO_2_ incubator. Only immediately before use, additional instable components are supplemented. These are: FBS or dialyzed FBS (Gibco, Thermo Fisher Scientific), *L*-glutamine, sodium pyruvate, as well as penicillin-streptomycin.

Table S2 and our cell culture media calculator “Media Minds,” both give an overview of all media that were used in this manuscript. Both contain a tab for complete/standard DMEM, as well as all DMEM variations (e.g., leaving out single amino acids). In addition, “Media Minds” enables calculating other cell culture media formulations, freely adjusting the medium to one’s needs.

Note: When using starvation media, it is crucial to use dialyzed FBS to avoid unintended re-supplementation of the intentionally removed components. In that case, the respective control medium should be the starvation medium to which the intentionally removed component is added again. Note: Labeled components can also be added after pH adjustment.

### The cell culture media calculator “Media Minds”

“Media Minds” translates the stock mixing strategy described in this manuscript into an interactive, platform-agnostic tool (Fig 1). “Media Minds” was written to encourage laboratories to formulate and adapt their own culture media. Given stock factors and a target volume, “Media Minds” automatically computes component volumes, back-fills water to reach the final volume, provides pH guidance for each workflow, and generates printable checklists; when an actual weighed mass is entered, adjusted dilution volumes are calculated in real time. It therefore complements and facilitates the wet-lab protocol by providing an automated and reproducible calculation layer.

**Table:** Implementation and distribution.

Feature	Description
Language stack	Plain HTML5, CSS3, and vanilla JavaScript. No external frameworks, plugins, or server-side code.
File structure	A single self-contained HTML file (MediaMinds.html, ∼120 kB, 3,072 lines of code) that includes markups, styles, data tables, and logic. Accessible via the following hyperlink: https://mediaminds.dkfz.de/
Runtime environment	Any modern standards-compliant browser (chromium > 88, Firefox > 85, Safari > 14, Edge > 88) on Windows, macOS or Linux. Mobile layouts are triggered by CSS media queries for screens <600px width.
Source control and DOI	Code is deposited/attached with the journal on submission.

#### Architecture overview

(1) **Navigation shell**—a responsive navigation bar changes four high-level pages: *Introduction*, *Medium Calculator*, *Help*, and *Contact*. Each page is switched via the helper showSection (id) function.
(2) **Data model**—all compounds, stock factors and preset protocols are hard-coded as JavaScript objects (getCompoundsForCategory(), appPresetsFA, silacSpecs, etc.). This approach guarantees that the scientific record always matches the executable code. The specific data structure for compounds includes molar mass, concentration, stock multipliers (stockX), stock molarity (stockM/stockmM), solvent, storage temperature, and notes.
(3) **Stoichiometric engine**—Core calculations use n = *c*⋅*V*; *m* = *M*⋅*n*, and *mg* = 1,000⋅*m*. To perform instant updates:
  • calcMassAuto/calcAdjAuto perform instant updates of mass and adjusted volume as the user types (stocks and individual compounds and custom media).
  • prefillCompoundVolume propagates suggested volumes from stock assembly tables into per-compound calculators.
  • assembleInOrg2, assembleSupplement1, assembleAminoAcids build 100×/10× stocks, compute component volumes, and back-fill water automatically.
  • updateInorgInst, updateSupp1Inst, updateAaInst update assembly instructions dynamically.
  • initAdditionalComponentsUI, updateACPrefills manage bottle-based volume calculations.
  • stdLoadPreset, varLoadPreset, appLoadPreset generate final-assembly checklists for standard DMEM, ingredients-based DMEM variants (with instant water volume updates via checkbox lists), and application-based DMEM presets (including Seahorse and SILAC).
  • stdLoadPreset varUpdateWater updates water requirements depending on the component volume.
(4) **Interface generation**—each calculator table is generated at runtime (createCalculator(), createCustomCalculator()). Results are written directly to elements so that copying or printing produces a clean, static record. The formatCompoundName() function automatically italicizes stereo-chemical prefixes (D-/L-) for proper chemical nomenclature display.
(5) **State management**—no frameworks are used; state is held in variables (selectedMedia, customCompoundCount, inStockPrepMode, etc.) and DOM data-attributes (e.g., data-vol). A page reload therefore resets the session. However, sessionStorage preserves the last visited section for 8 h using saveCalculatorState() and restoreCalculatorState() functions, helping users resume work after accidental refreshes.
(6) **Printing and reset**—printAllData clones the current view, converts inputs to static values, and opens a print-friendly page. resetAllCalculators clears calculated fields without reloading. In addition, the print view uses cloneNode(true) to preserve the exact state.
(7) **Validation**—Outputs from “Media Minds” were cross-checked against manual calculations for representative recipes, including complete DMEM, Met-free DMEM, Seahorse DMEM, and a six-step SILAC nascent-proteome series. The calculator includes input validation via sanitizeNumericValue() and validateNumericInput() functions to prevent calculation errors from invalid entries. All values were within the weighing precision of a calibrated 0.1 mg analytical balance (Kern and Sohn).

The application runs entirely client-side with no external dependencies, network requests, or data collection. All calculations occur locally in the browser, ensuring complete privacy of research formulations.

#### How to use the calculator (bench workflow)

This section provides a step-by-step guide to every part of the Media Minds calculator, so you can prepare custom media easily, consistently, and reproducibly. The application is a single-file web app accessible through https://mediaminds.dkfz.de/ that opens locally in the browser and runs once loaded.

To get started, open https://mediaminds.dkfz.de/ in any modern browser (Chrome, Firefox, Safari, or Edge are all supported). The main page will appear immediately, with the title (“Media Minds v_1.0”) and a subtitle at the top. Just below, there is a navigation bar with several sections: Introduction, Medium Calculator, Help (with sub-options for How to use, Tips and Tricks, and Contact).

For an overview of what the tool does, click the Introduction button. This section explains the purpose of Media Minds and why it is useful. The Help dropdown menu provides detailed assistance:

• “How to use” includes a step-by-step guide.
• “Tips and Tricks” shares best practices (like labeling stocks).

#### Accessing the Medium Calculator

To prepare media, click the “Go to Medium Calculator” button or select Medium Calculator from the navigation bar. This brings you to a new page with three main modules: (1) stock preparation, (2) assemble DMEM (standard DMEM and DMEM variations), and (3) individual compounds and custom media. Each module includes a title and a brief description.

(1) Stock Preparation (Module 1)

Start by choosing “Stock Preparation.” Here, you can select which stock to prepare: 100× Inorganic Salts, 100× Salts and Vitamins, 10× Amino Acids, or Additional Components. Additional components includes two subsections: (1) single components to weigh in (sodium chloride, sodium bicarbonate, riboflavin, folic acid) with a “Bottles to supply” calculator that prefills volumes for multiple DMEM bottles, and (2) store-bought solutions listed for reference. After your selection, enter the desired final stock volume (in milliliters); component volumes, and the mixing instructions update automatically.

A clear table will show each component, required volumes, and the amount of water needed to add to reach the target stock concentration. Linked calculators for individual components are pre-filled with suggested volumes and compute mass (g/mg) instantly; if you type the actual weighed amount (mg), the adjusted dilution volume is recalculated automatically to preserve the exact molarity. Instructions beneath the table walk you through mixing, labeling, and storing your stock (e.g., at 4°C).

(2) Making Standard DMEM or DMEM Variations (Module 2)

Selecting “Standard DMEM” lets you enter the final volume you want (e.g., 500 ml). The page displays five clearly labeled sections: Initial additions, inorganic salts, salts and vitamins, amino acids and additional components. Each section expands to show exactly how much stock solution or water to add.

There is also a table for pH adjustments, so you can record your starting pH, any HCl added, and your final pH. Standard DMEM and most variants target pH 8.1–8.2; Seahorse medium targets pH 7.4. Seahorse medium includes a special note about pH drift and potential NaOH correction due to lack of buffering. Nutrient depletion protocols (NAM-free, Met-free, Trp-free) display a reminder to prepare control media with the omitted nutrient included. Below that, a checklist helps track post-pH supplements (such as (dialyzed) FBS, sodium pyruvate, glutamine, and antibiotics) with the indicated volume. You can reset or print the final recipe as needed.

Under “DMEM Variations,” you can either choose by application (e.g., Seahorse, NAM-free, Met-free, Trp-free, SILAC, or Trp tracing) or by Ingredients (salts and vitamins, amino acids, or inorganic salts). SILAC contains six sub-options for treatment C/T, pre-labeling C/T, and SILAC C/T.

For the application workflow, select your application, enter the final volume, and the tool automatically updates the five recipe sections. Adjust pH and supplements, then print or reset the recipe.

For the ingredients workflow, choose the modification type (e.g., modify all), enter the final volume, and select exactly which components to include. Water volume updates automatically as you adjust your choices. Once your recipe is finalized and pH is set, you can print your protocol.

(3) Individual Compounds and Custom Media (Module 3)

If you need a fully customized compound formulation, select “Individual Compounds and Custom Media.” This opens the Custom Compounds interface, where you enter each compound’s name, molar mass (g·mol^−1^), desired concentration (mol·l^−1^), and final volume (ml). Mass (g and mg) is computed instantly as you type; when you enter the actual weighed mass (mg), the adjusted dilution volume (ml) is recalculated automatically. If the actual weighed amount differs, you can input it and recalculate the adjusted volume.

You can add up to 30 compounds and generate a clear, printer-friendly summary with the “Print All Data” button. This option will help you to calculate the formulation of any compound you require, whether it is for media preparation or routine lab work dilutions.

#### Printing

All recipe and formulation pages include a print option. Clicking “Print” opens a simplified layout for easy reading at the bench, PDF export, or direct printing. The print view converts inputs to static values so your checklist reflects exactly what was entered.

#### Helpful reminders

If you ever get lost, use the “Back” buttons at the top of each section to navigate. For quick access to instructions, consider bookmarking the “How to use” page or printing a small test batch protocol first when trying a new formulation.

In addition, the calculator includes several features to enhance your workflow: It automatically remembers your last location for 8 h in the same browser tab. Keyboard shortcuts are available—press Esc to go back to the previous screen, or Ctrl/Cmd+P to open the print dialog directly. When working with stock preparations, suggested volumes appear with a yellow background in the input fields; clicking in any field removes the suggestion so you can enter your own values. These visual and functional cues help streamline media preparation, especially during repetitive calculations.

This walkthrough is designed so that everyone—from beginners to experienced researchers—can navigate and use Media Minds confidently to produce reliable results.

### Custom media for metabolic studies—Seahorse medium

Measuring cellular energetics and metabolism by Seahorse XF technology requires using specialized Seahorse DMEM that is free of certain components that can interfere with the measurements (Fig 3A–D). The main differences between standard DMEM and Seahorse DMEM are the exclusion of glucose, sodium bicarbonate, *L*-glutamine, sodium pyruvate, penicillin-streptomycin and FBS. The absence of sodium bicarbonate and FBS is particularly important because they would change the buffering capacity of the media, leading to inaccurate pH measurements during the assay.

Seahorse DMEM was prepared in the same way as complete DMEM, except that glucose, sodium bicarbonate, *L*-glutamine, sodium pyruvate, penicillin-streptomycin and FBS were omitted (Table S1, “Seahorse DMEM”). Before use, the pH was adjusted to 7.4 using HCl. Note: Ensuring the pH is precisely adjusted to pH 7.4 at 37°C is critical for the accuracy of the Seahorse assay, as even small pH fluctuations can significantly affect cellular respiration measurements. As no buffer system is contained in the Seahorse DMEM, pH shifts may occur more rapidly and drastically compared with complete DMEM. Hence, it might also be necessary to adjust the final pH with 1 M NaOH.

Shortly before performing the Seahorse assay, the Seahorse DMEM was supplemented with 1 g/liter or 4.5 g/liter glucose (Sigma-Aldrich), 1 mM sodium pyruvate and 2 mM *L*-glutamine. Cells were washed with PBS and glucose-containing Seahorse DMEM was added to the cells for 3 h.

### Preparation of commercial Met-free DMEM

In addition to self-made Met-free DMEM, also commercially available, amino acid-free cell culture medium (Genaxxon) was used for experiments, indicated by black dots in Fig 5A and C–E. The commercial amino acid-free medium was supplemented such that it finally contained all single amino acids (using the individual 1,000x or 100x amino acid stocks) except Met. The respective control medium was supplemented with Met. In addition, the media were supplemented with dialyzed FBS, penicillin-streptomycin and 3.5 g/liter glucose (as the commercial medium already contains 1 g/liter glucose). Note: Be aware that the commercial amino acid-free DMEM used for the generation of Met-free medium additionally contains phenol red, which may affect downstream assays sensitive to this pH indicator.

### Nutrient depletion experiments

To study the intracellular effects of NAM (Fig 4B–D) and Trp starvation (Fig 6B) for 24 h, 1.2 × 10^4^ cells were seeded in 12-well plates (TPP). For 48 h of Met (Fig 5B–E) or Trp deprivation (Fig 6C–E), either 7.5–8.0 × 10^5^ or 3.2 × 10^5^ cells were plated in 10 cm (TPP) or 6 cm (Greiner Bio-One) cell culture dishes, respectively. Cells were allowed to attach overnight in commercial DMEM supplemented with 10% FBS, 1 mM sodium pyruvate, and 2 mM *L*-glutamine. On the following day, cells were washed once with PBS to remove residual seeding medium and then provided with the respective starvation medium (NAM-free, Trp-free, and Met-free). All starvation media were supplemented with 10% dialyzed FBS. Cells were harvested for subsequent cellular assays after 24 or 48 h in the respective starvation media.

### Cell doubling measurements using sulforhodamine B assay

Assessment of cellular proliferation was conducted using the Sulforhodamine B (SRB) assay, following the procedure outlined by Vichai and Kirtikara (2006) (Fig 2B). This assay quantifies cellular protein content by staining with SRB at acidic pH.

To assess proliferation rates, LN-229 and G-142 cells were cultured in both commercial DMEM and self-made DMEM. Cells were seeded at 2 × 10^3^ cells/cm^2^ (three technical replicates per condition) in pre-warmed 24-well plates to ensure even distribution across the entire well. When seeding, PBS was pipetted between the wells to promote a more humidified atmosphere. The cell suspension was then added slowly along the wall of each well. Baseline (day 0) optical density (OD) measurements were acquired immediately after seeding to calculate doubling rates and account for plating efficiency, as described below.

Cells were allowed to grow until they reached confluency. Cells were then fixed with 10% trichloroacetic acid (TCA) (Carl Roth), incubated for 1 h at 4°C and washed with tap water three times. Plates were dried overnight and then stained with 0.057% (wt/vol) SRB (Sigma-Aldrich) in 1% acetic acid (Carl Roth) for 30 min at RT. To prevent any cells from detaching, the fixing and staining solution was slowly added along the walls of the well. Note: Since SRB is light-sensitive, either switch off the light or cover the plates during this incubation period. After staining, cells were washed with 1% acetic acid to remove excess dye. Gently tap the plate on a paper towel between washes to remove any residual stain as this may lead to an increased background signal and affect the accuracy of the OD measurements.

After drying overnight, the dried stain was then solubilized in 10 mM Trizma base (pH 10.5; Sigma-Aldrich). To ensure complete solution of the stain, the plate was placed on an orbital shaker for 5 min or incubated for a total of 15 min with shaking every 5 min. Finally, OD measurements of the solubilized dye were performed using a CLARIOstar microplate reader (BMG Labtech) with an excitation wavelength of 488 nm and an emission wavelength of 585 nm to determine cell numbers.

This method allowed clear visualization of relative cellular proliferation rates across experimental days. The assay principle is based on the fact that more cells will elicit a higher OD value. The relative cell number doubling time was finally calculated by dividing the OD values at the respective days (N_t_) by the OD value at day 0 (N_0_). These values were then log_2_-transformed and compared for cells cultured in commercial DMEM versus self-made DMEM.

In detail, the OD measurements were analyzed as follows: first, for each experimental condition at each time point, the average OD was calculated from three technical replicates:

$${\text{OD}}_{\text{avg},\;\text{day}\;n}=\frac{{\text{OD}}_{\text{replicate}\;1}+{\text{OD}}_{\text{replicate}\;2}+{\text{OD}}_{\text{replicate}\;3}}{3}$$ (1)

Next, each day’s average OD was normalized to the baseline (day 0) OD, representing the initial cell density immediately after medium replacement and TCA fixation:

$$\text{Normalized}\;\text{fold}\;\text{change}\;\left(\text{day}\;n\right)=\frac{{\text{OD}}_{\text{avg},\;\text{day}\;n}}{{\text{OD}}_{\text{avg},\;\text{day}\;0}}$$ (2)

Finally, the normalized fold change values were converted, with the log_2_ fold change value of day 0 set by default to zero to provide an intuitive measure of the cell number increase over time:

$$\log_{2}\;\text{fold}\;\text{change}\;\left(\text{day}\;n\right)=\log_{2}\left(\frac{{\text{OD}}_{\text{avg},\;\text{day}\;n}}{{\text{OD}}_{\text{avg},\;\text{day}\;0}}\right)$$ (3)

### Microscopy

#### Bright field tilescan of SRB-stained plates

SRB-stained 24-well plates were imaged on the Leica MICA Microhub as transmitted-light images using a 1.6× HC PL FLUOTAR dry objective with a numerical aperture (NA) of 0.05 and a working distance of 3.4 mm (Fig 2A and C). Exposure and illumination settings were kept constant within each experiment. To capture a contiguous 4 × 4 block of wells, we drew a single rectangular ROI around the well centers using the rectangle ROI tool. Before imaging, we ran the high-speed AF “Image-Based” autofocus once per ROI to compensate for plate-height differences. Stage tile scanning was configured with four rows, four columns and a fill factor of 5% overlap, resulting in acquisition of ∼154 individual frames per block that were later stitched. Frames were automatically stitched in LAS X to generate a high-resolution montage for each plate. For each cell line (LN-229 and G-142), we imaged days 0–7 for n = 4 biological replicates; each condition (commercial DMEM versus self-made DMEM) included n = 4–6 technical replicates (separate wells) per plate.

**Figure S2.**
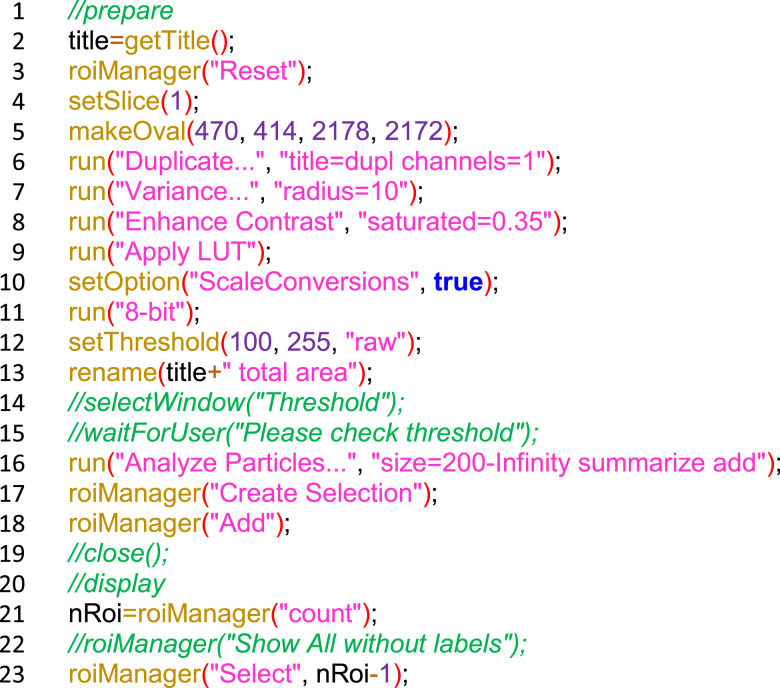
ImageJ macro for SRB signal quantification from transmitted-light images of 24-well plates.

Montages were opened in ImageJ (v1.53), split into RGB channels. The red channel (SRB signal) was then analyzed per well using a custom-developed macro in ImageJ (Fig S2). In short, we duplicated the red channel, applied a variance filter (radius 10) and contrast enhancement to reduce uneven background, converted the image to 8-bit, used a threshold of 100–255, used the analyze particles function to isolate each well interior, and measured the integrated pixel density (sum of pixel intensities) for each well reflecting relative cell number. Results were exported as CSV files.

#### Visualization of cell morphology using wide field microscopy

The morphology of LN-229 and G-142 cells cultured in either commercial or self-made DMEM was captured by microscopy using the Leica MICA Microhub (Fig 2D and E). Transmitted-light images were processed for publication with the open-source Open Microscopy Environment Remote Objects (OMERO) software platform.

Specifically, living, unfixed LN-229 and G-142 cells were imaged with the MICA system. Cells were cultured either in standard 24-well round, transparent plastic plates (Greiner Bio-One) or in μ-Plate 24-well plates (ibidi GmbH), which have a flat, optically clear polymer coverslip bottom and black walls for high-resolution imaging. Imaging was performed with the sample in air (refractive index, n = 1,000) using a 10× HC PL FLUOTAR dry objective with a numerical aperture (NA) of 0.32 and a working distance of 11.2 mm. For each well, 26 images were acquired from independent fields of view at predefined, consistent positions, focusing on the central area of each well. For each experimental condition, four to six wells were analyzed, yielding a total of 104–156 images per condition per day.

Transmitted-light images were acquired in integrated modulation contrast (IMC) mode with the following settings: offset 0, light intensity 128, exposure time 40 ms, and gain 1.0. Images were captured at high resolution (2,432 × 2,032 pixels), corresponding to a field of view of 1.398 × 1.168 mm and a pixel size of 575 nm. All imaging parameters were kept constant across all wells within each experimental session.

**Figure S3.**
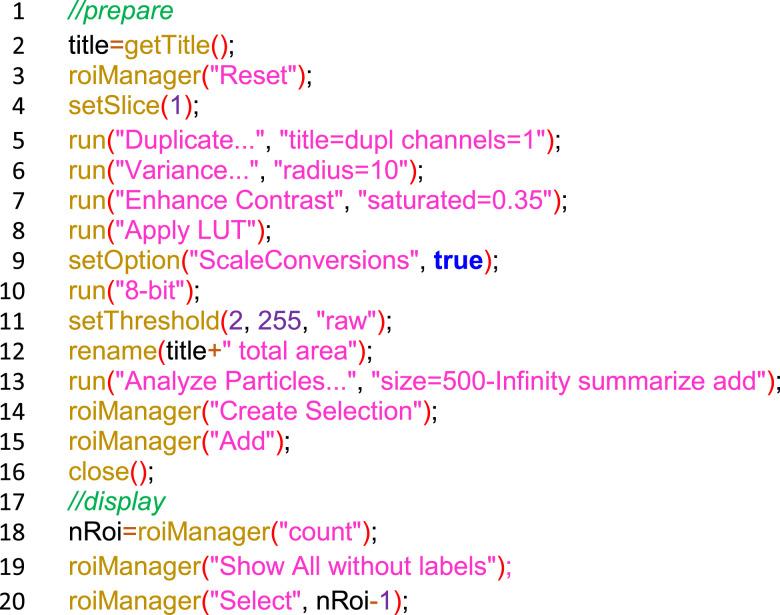
ImageJ macro for cell density quantification from transmitted-light images.

Images were analyzed using a custom-developed macro in ImageJ to calculate the percentage of cell-covered area per image, as described in Fig S3. Before analysis, images with artifacts such as debris, uneven illumination, or interference from plate texture were excluded (between 5% and 10% per condition).

Representative IMC images were prepared for publication with OMERO.web (v5.29.0), specifically using the OMERO.figure tool. Images were displayed at 200% zoom (rescaled pixel dimensions), resulting in a displayed field of view of 1,216 × 1,016 pixels, corresponding to a field of view of 698.8 × 584 μm. The scale bar (250 μm) was automatically generated from image metadata to reflect true spatial dimensions. All presented images per cell line were acquired from the same location on the same plate at different time points.

### Assessment of metabolic functions by Seahorse XF

**Figure S4.**
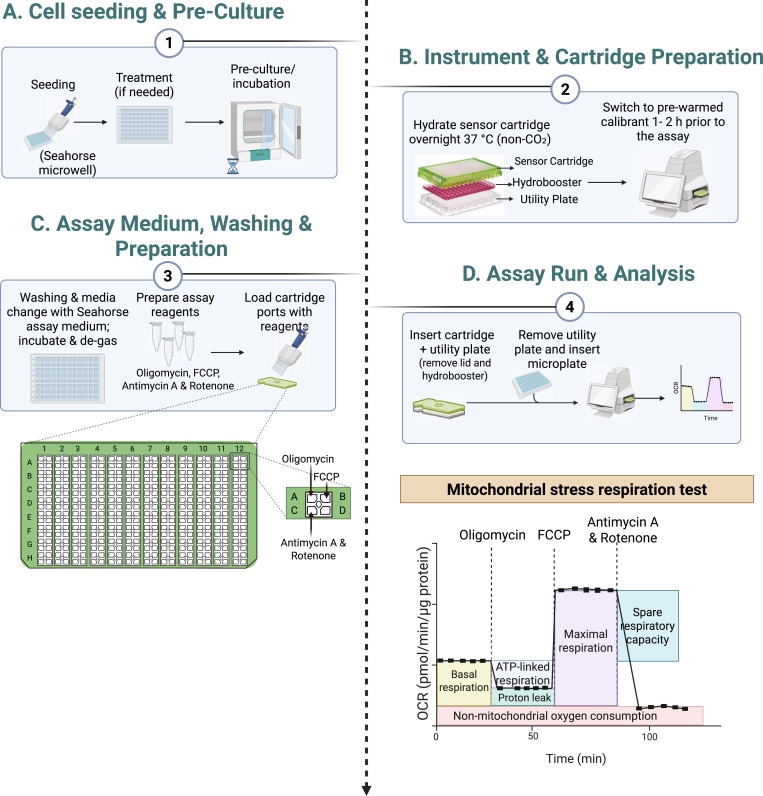
Simplified scheme of the workflow for a mitochondrial stress respiration test with the Seahorse XF analyzer. **(A, B, C, D)** represent consecutive steps of the workflow.

Seahorse experiments were performed according to the manufacturer’s recommendations (Figs 3A–D and S4). For further explanation, visit the Agilent homepage.

#### Cell seeding and pre-culture

(1) Optimize Seeding Density
  LN-229 and G-142 cells were seeded at 2 × 10^5^ cells/ml (100 μl per well), resulting in 20,000 cells per well in DMEM in a Seahorse XF96 cell culture microplate (Agilent) so that they reach optimal confluency within 48 h.
(2) Distribute Cells Evenly
  After adding the cell suspension to each well, let the plate rest at RT for ∼1 h in the tissue culture hood. This helps cells settle uniformly and reduces edge effects (Lundholt et al, 2003; Yang et al, 2021).
  Transfer the plate to a 37°C, 5% CO_2_ incubator (SANYO Electric Co., Ltd.) and allow cells to attach for 12–18 h (or overnight).
(3) Include Background Correction Wells
  Reserve four wells (A1, A12, H1, H12) with medium only (no cells) for background correction. This ensures that any baseline signal is accounted for during data analysis.

#### Instrument and cartridge preparation

(4) Hydrate the Sensor Cartridge
  Hydrate the sensor cartridge in the Seahorse utility plate (Agilent) with Seahorse calibrant (Agilent). Use autoclaved water or sterile water to hydrate the sensor cartridge in the Seahorse utility plate (Agilent) overnight at 37°C in a non-CO_2_ incubator (Heraeus) and switch to pre-warmed Seahorse calibrant 1–2 h before the assay.
  Hydrate maximum 3 d in advance. If hydration exceeds 6 h, seal the utility plate with tape or parafilm to prevent evaporation. On the day of the assay, gently remove the cartridge from the incubator, placing it upside down on a clean surface, avoid touching the sensors.
(5) Turn on the Seahorse XF Analyzer
  Switch on the instrument, the connected computer, and the non-CO_2_ incubator at least 5 h before starting the assay.
  Open the Wave software, click “Heater on,” and ensure the temperature is set to 37°C.
  Wait until the lower-left “connected” icon in Wave turns green to confirm stable communication with the instrument.

#### Assay medium, washing, and preparation of assay

(6) Prepare Seahorse Assay Medium
  Use custom-made Seahorse DMEM to avoid buffering the media (Table S2).
  Check carefully whether the pH is still 7.4 (using a pH meter [inoLab]) as small deviations can drastically affect cellular metabolism.
(7) Wash the Cells
  On the day of the assay, retrieve the plate from the CO_2_ incubator (SANYO Electric Co., Ltd.).
  Gently remove the old culture medium, leaving ∼20 μl behind to avoid disturbing the cell monolayer.
  Add 180 μl of the Seahorse assay medium to each well and repeat this wash step three times.
(8) De-gas the Plate
  Place the washed plate in a non-CO_2_, 37°C incubator for 1 h to allow the cells to equilibrate.
  This de-gassing period is critical to stabilize the medium’s pH and temperature before the measurement begins.
(9) Load Calibrant for Cartridge Calibration
  Just before the assay, load 200 μl of fresh Seahorse calibrant into each well of the utility plate.
  Re-insert the sensor cartridge and incubate at 37°C for 45–60 min. This step allows the cartridge to calibrate before measuring your samples.
(10) Load the Compounds
  Prepare the injection ports in the hydrated sensor cartridge with the following compounds (final well concentrations):
    ⁃1 μM oligomycin A (to inhibit ATP synthase)
    ⁃0.5 μM FCCP (to uncouple oxidative phosphorylation)
    ⁃0.5 μM antimycin A and 0.5 μM rotenone mix (to block electron transport)
  The inhibitor concentrations (oligomycin A, FCCP, antimycin A and rotenone) are empirically optimized in preliminary dose-response experiments to ensure effective pathway inhibition without nonspecific toxicity.

#### Running the Seahorse XF assay, data collection, and analysis

(11) Calibrate and Start the Measurement
  Load the cell plate into the Seahorse XFe96 Analyzer.
  Place the sensor cartridge on top, and follow the Seahorse XF Wave Analyzer software (Agilent) prompts to complete calibration. Remove lid and hydrobooster.
  If there is a problem with the recognition of the barcode, re-read the plate. Otherwise, choose to type in manually the barcode.
  Once calibration is done, the cartridge will automatically lower onto the cell plate, and the assay will start.
  After the run, verify whether the cartridge ports are empty at the end of the run. If the ports are not empty, there could be a problem with the manifold (sealing of injection ports).
(12) MitoStress Test Protocol
  The analyzer typically cycles through mixing (5 min), waiting (5 min), and measuring (5 min) phases.
  Check if the cells require more time to adapt to the new medium. If the peaks and shifts after each drug addiction are clearly visible, the adaptation period is likely sufficient. If the traces are noisy, flat, or responses are blunted, consider increasing the adaptation time to allow the cells to equilibrate in the new medium before starting the assay.
  Monitor OCR in real time to see how cells respond to each inhibitor.
(13) Collecting Data with Wave Software
  The OCR measurement is recorded in the Seahorse XF Wave Analyzer software.
  Ensure background signals from the empty wells (A1, A12, H1, H12) are subtracted appropriately. Even small deviations in background can alter your final data.
(14) Normalization
  OCR values are normalized to total protein content immediately post-assay to account for inter-well variability.

Finally, OCR values were plotted as a curve (Fig 3B) or as bar graphs (Fig 3C and D). Fig 3C depicts the mean OCR value at the final measurement of each intervention phase (when the effect had stabilized) and includes basal respiration, ATP-linked respiration, maximal respiratory capacity, and spare respiratory capacity. Fig 3D displays the average OCR value across the entire measurement time of 125 min. Data are depicted as mean ± SEM of n = 3 biological replicates, each consisting of at least n = 6 technical replicates. In total, at least 31 individual data points for each intervention were measured.

### Liquid chromatography (LC)—mass spectrometry (MS) methods

For robust metabolite measurements, it is crucial to keep the following key considerations in mind (see also Box 1):

• Work on ice or snap freeze samples immediately to avoid degradation of rather instable metabolites while harvesting. Perform metabolite extraction as quickly as possible and on ice using cooled solvents. Try to avoid repeated freeze-thaw cycles. In general, ensure that your sample preparation is adapted to instable metabolites.
• Measure both intra- and extracellular metabolite concentrations and also collect pure media samples to measure the metabolites of interest in the media as baseline for the cell-dependent measurements.
• Use heavy-labeled standards and check retention time and MS spectra for identification of compounds and absolute quantification when needed. Dissolve all standards in MS-compatible solvents. Try to avoid any harsh solvents such as DMSO during sample preparation for metabolite measurements or for dissolving of pure standards as these can cause high background signals during your LC-MS/MS measurements.
• Always normalize metabolic data to cell number or total protein to avoid artifacts due to cell proliferation differences. Different extraction methods could potentially interfere with protein measurements (background) or cell counting (clumped cells). In that case, consider using satellite plates with the exact number of cells and treatment that can be used solely for normalization.
• Use non-autoclaved tubes and filter tips to avoid artifacts due to autoclaving.
• Only use LC-MS-grade solutions as lower purities can affect the instruments and your measurements.

We provide information about the multi-reaction monitoring (MRM) parameters in the source table under “MRM paramenters.”

#### Metabolite extraction protocol for nicotinamide-free medium and tryptophan-tracing experiments

For assessment of NAM in self-made complete and NAM-free media (Fig 4A) and for Trp-tracing experiments (Fig 6G and H), samples were analyzed by LC-MS according to the following protocol: metabolites were extracted from cells seeded in 6-well plates (Greiner Bio-One), with densities adjusted to standardized growth rates across experimental conditions as already shown (Ghrayeb et al, 2024). Each LC-MS batch incorporated blank runs, pooled quality-control samples, and ISTDs to ensure reproducibility and detection sensitivity. Across experiments we analyzed n ≥ 4 technical wells per condition within each biological replicate.

For measurement of the NAM medium content (Fig 4A), complete and NAM-free medium was placed in plates (without cells) and kept in the incubator for 72 h.

For Trp-tracing experiments (Fig 6G and H), LN-229 and A-172 cells were seeded at 1.5 × 10^5^ cells per well (6-well plate; 2 ml medium per well, corresponding to 7.5 × 10^4^ cells/ml) in commercial, complete DMEM. After 24 h, a medium change to complete self-made DMEM was performed. After 48 h, medium was again replaced with self-made DMEM containing either unlabeled Trp (#T8941; Sigma-Aldrich) as in the normal self-made DMEM, or labeled Trp (Trp-^13^C_11_,^15^N_2_) (#574597; Sigma-Aldrich), for a 24 h pulse, after which metabolites were extracted and isotopologue abundances were quantified. The self-made medium contained dialyzed FBS to avoid indirect re-supplementation of Trp.

An extraction solvent mixture consisting of methanol (Merck Millipore), acetonitrile (Merck Millipore), and water (LC-MS grade, Merck Millipore) in a 5:3:2 (vol/vol/vol) ratio by volume, pre-chilled to −20°C, was used for extraction. For pure medium samples (Fig 4A), 20–25 μl of medium was mixed with 980 μl of cold extraction solvent, vortexed, incubated on a shaker with 3.29*g* at 4°C for 10 min, and centrifuged at 16,100*g* for 10 min at 0–4°C. Supernatant was then transferred into new reaction tubes that were stored at −80°C until LC-MS analysis. Before analysis, supernatants were transferred to glass HPLC vials (Agilent).

Intracellular metabolites (Fig 6G and H) were extracted by first discarding the cell supernatants and washing the cells on ice with pre-cooled PBS to remove residual medium. Then 600 μl of cold extraction solvent were added to the cells in the well. The six-well plates were agitated for 10 min at 0–4°C to facilitate thorough metabolite extraction. The extraction solvent was then transferred to a reaction tube and centrifuged at 16,100*g* for 10 min at 0–4°C. Supernatants were transferred into new reaction tubes and were stored at −80°C until analysis. Before analysis, supernatants were transferred to glass HPLC vials.

Metabolomic profiling was performed using a Dionex Ultimate UPLC system (Thermo Fisher Scientific) coupled to a Q Exactive Orbitrap mass spectrometer (Thermo Fisher Scientific). A ZIC-pHILIC column with guard column (Merck Millipore) facilitated the chromatographic separation under a gradient from 20% aqueous phase (20 mM ammonium carbonate [Thermo Fisher Scientific], pH 9.2 adjusted with 0.1% ammonium hydroxide [Thermo Fisher Scientific]) to 80% acetonitrile. Mass spectrometric detection was executed in polarity switching mode, capturing a mass range from 67 to 1,000 *m/z* at a resolution of 35,000 at 200 *m/z*.

NAM, Trp, Kyn, as well as the isotopically labeled forms of Trp and Kyn (Trp-^13^C_11_,^15^N_2_ and Kyn-^13^C_10_,^15^N_2_) were identified by matching exact masses and retention times against an in-house library of standards.

For the measurement of NAM, the following parameters were identified: RT = 2.59 min, *m/z* = 123.05529, formula C_6_H_6_N_2_O. For targeted metabolomics, unlabeled Trp (RT = 4.54 min, *m/z* = 205.09715, formula C_11_H_12_N_2_O_2_), labeled Trp (RT = 4.54 min, *m/z* = 218.12813, formula ^13^C_11_H_12_^15^N_2_O_2_), unlabeled Kyn (RT = 3.81 min, *m/z* = 209.09207, formula C_10_H_12_N_2_O_3_), and labeled Kyn (RT = 3.81 min, *m/z* = 221.11969, formula ^13^C_10_H_12_^15^N_2_O_3_) were measured and confirmed. Metabolite identities were confirmed by matching retention times, exact masses, and MS fragmentation patterns to authenticated, pure standards. Peak areas were normalized to cell number, with data acquisition and peak analysis managed by Thermo Xcalibur (Thermo Fisher Scientific) and Thermo TraceFinder software (Thermo Fisher Scientific), respectively. Visualization of metabolite data was performed using Metabolite-Auto Plotter 2.6 software to facilitate interpretation (Pietzke & Vazquez, 2020).

#### Metabolite extraction protocol for assessment of intracellular NAD^+^ and NAD^+^-related metabolites

For assessment of intracellular Trp, NAM, M-NAM, and NAD^+^ in self-made DMEM (Figs 4B–D and 6B) samples were analyzed by LC-MS as already published (Høyland et al, 2024). Cells cultured with or without NAM for 24 h were twice washed with ice-cold PBS and quickly detached from the surface by adding ice-cold 80% (vol/vol) LC-MS grade methanol and scraping with a clean rubber spatula. Samples were incubated on a spinning wheel for 30 min at 4°C and subsequently centrifuged at full-speed and 4°C for 15 min. Supernatants were immediately frozen in liquid nitrogen and samples were kept at −80°C until further processing and analysis.

##### Generation of ^13^C-^18^O-labeled standard from HeLa S3 cells

^13^C-^18^O-labeled standard was generated in HeLa S3 cells as already published (Høyland et al, 2024). Three million HeLa S3 cells were seeded in complete DMEM in 15 cm plates (TPP). The next day, the medium was removed and cells were washed twice with PBS and incubated with custom-made glucose-, pyruvate-, *L*-glutamine-, pantothenate-, NAM-, and phenol red-free DMEM (Cell Culture Technologies) supplemented with dialyzed FBS, *L*-glutamine, penicillin-streptomycin, as well as 33 μM ^18^O-Nam (Migaud lab, synthesized as described previously [Makarov et al, 2019]), and 25 mM *D*-glucose-^13^C_6_ (Cambridge Isotope Laboratories Inc.). The medium was renewed after 24 h. After 48 h of incubation with the labeled compounds, the medium was removed; the cells were washed twice with PBS and lysed in 8 ml ice-cold 80% (vol/vol) LC-MS grade methanol. The cells were detached from the plates using a cell scraper and the samples were transferred to 50 ml tubes. To ensure full transfer, the plates were washed with 5 ml ice-cold 80% (vol/vol) LC-MS grade methanol again. The samples were subsequently frozen at −80°C. After overnight incubation at −80°C, the samples were vortexed for 20 s, and centrifuged at 4°C and 3,000*g* for 10 min. The supernatants were transferred to fresh Eppendorf tubes and the residual cell pellets were discarded.

##### Triphasic extraction

500 μl of cell lysate were mixed with 300 μl MilliQ H_2_O, 40 μl of isotope labeled ISTD (^13^C-^18^O labeled standard) and 500 μl chloroform (Sigma-Aldrich). For medium samples, 500 μl of sample were mixed with 300 μl MilliQ H_2_O, and 500 μl chloroform. The samples were vortexed for 10 s and centrifuged for 30 min at 16,000*g* and 4°C. 550 μl of the upper phase were removed, dried, and dissolved in 80% (vol/vol) methanol enabling a higher sample concentration by a factor of 11.

##### LC-MS analysis

Analysis was conducted on a Dionex UltiMate 3000 liquid chromatography system (UPLC) interfaced with a Q Exactive mass spectrometer equipped with an electrospray ionization source (ESI) from Thermo Fisher Scientific. Analytes were separated on an Atlantis Premier BEH Z-HILIC 2.5 μm VanGuard FIT 2.1 × 100 mm column (Waters) which was kept at 40°C during the analysis. The total run time for each injection was 18 min and the injection volume was 10 μl. Separation was done at a flow rate of 0.3 ml/min. Solvent A and B were 3% and 90% acetonitrile (Sigma-Aldrich), respectively, both buffered with 10 mM ammonium acetate. The gradient (B) composition was: 94.4–84% over 5 min, 84–58.6% over 7 min, 30% for 4 min, followed by 2 min at 94.4%. Heated ESI was conducted using the positive ion polarity mode, and a spray voltage of 3.5 kV. The sheath gas flow rate was 48 units with an auxiliary gas flow rate of 11 units, and a sweep gas flow rate of 2 units. The capillary temperature was 256°C and the auxiliary gas heater temperature was 413°C. The stacked-ring ion guide (S-lens) radio frequency (RF) level was at 30 units. Automatic gain control was set to 2 × 10^5^ ions and the maximum injection time was 200 ms. Ions were monitored in positive Full MS and targeted single ion monitoring (t-SIM) modes. The Full MS and t-SIM resolution was 70,000 at *m/z* = 200. The Full MS spectra ranges were 80–400 and 400–1,000 *m/z*. Accurate mass quantification was performed using Thermo Xcalibur. All retention times were verified by injection of the pure compounds. For NAD^+^, NAM, and M-NAM, metabolite contents were determined on the basis of the labeled ISTD and are given in nmol/mg protein. Protein content was determined using Pierce BCA Protein Assay Kit (Thermo Fisher Scientific).

#### Metabolite extraction protocol for methionine- and tryptophan-free media

For assessment of Met (Fig 5A) and Trp (Fig 6A) in self-made DMEM, samples were analyzed by LC-MS/MS according to the following protocol. Self-made Met-free, Trp-free, and complete DMEM were directly frozen in liquid nitrogen (without being in touch with cells). Samples were kept at −80°C until further processing and analysis.

Samples were processed with fully heavy-labeled Trp (Trp-^13^C_11_,^15^N_2_) or Met (Met-^13^C_5_,^15^N; Sigma-Aldrich) as ISTD. For the extraction of metabolites, samples were first thoroughly mixed with ice-cold, ISTD-containing 80% (vol/vol) MeOH and further extracted at −20°C for 30 min. To remove cell debris, samples were centrifuged at maximal speed for 15 min at 4°C. The supernatants were dried in a pre-cooled SpeedVac (SRF110; Thermo Fisher Scientific) and stored at −80°C until further processing.

For LC-MS/MS measurements, dried samples were thoroughly resuspended in 50 μl acidified mobile phase (0.2% formic acid [FA, Biosolve], 1% acetonitrile [Merck Millipore] in HPLC-H_2_O [Merck Millipore]) and centrifuged at maximal speed for 15 min at 4°C. 35 μl of each supernatant was transferred to Truview total recovery glass vials (Waters). The samples were injected into an ultra-high-performance liquid chromatography (UHPLC) system (Acquity Waters Premier System, Waters) coupled with an ESI source to a high resolution time of flight (TOF) mass spectrometer (Zeno-TOF 7600 System; AB SCIEX Germany GmbH). Samples were loaded onto an analytical C18-column (Acquity HSS T3 C18 column VanGuard 1.8 μm, 2.1 × 150 mm; Waters) operating at 35°C and using a flow of 0.45 ml/min. Metabolites were separated by a gradient of 1–99% solvent B in 20 min (Solvent A: 0.1% FA in HPLC-H_2_O; solvent B: 0.1% FA in 100% acetonitrile) as follows: 1.5 min 1% B, 6 min 15% B, 14 min 80% B, 15 min 99% B for 2 min followed by returning back to 1% B until 20 min. Mass spectra of Trp and Met were measured in positive mode by using multiple reaction monitoring with high resolution (MRM-HR). For MS spectra, the collision energy (CE) was set to 10 V with a declustering potential (DP) of 40 V, while for MS/MS spectra the CE and DP values for Met and Trp were determined using a separate-guided MRM measurement. Analysis of the results was performed using the Sciex OS (v.3.3.0) Software (AB SCIEX Germany GmbH).

### Protein isolation and immunoblot

Protein isolation and immunoblots were performed as previously described in Prentzell et al (2021). Total proteins were isolated and used to test the abundance of proteins and their posttranslational modifications. All steps were performed on ice to prevent protein degradation. First, cells were once washed with PBS and subsequently lysed in RIPA buffer (1% IGEPAL CA-630 [Sigma-Aldrich], 0.1% sodium dodecyl sulfate [SDS, Carl Roth], and 0.5% sodium deoxycholate [AppliChem] in PBS) containing freshly added phosphatase inhibitor cocktails 2 and 3 (Sigma-Aldrich) and complete protease inhibitor cocktail (Roche). After centrifugation for 10 min at 13,000*g* and 4°C, the supernatant was collected, and the protein concentration was determined at 595 nm using the Protein Assay Dye Reagent Concentrate (Bio-Rad) on a GE Healthcare spectrophotometer. To ensure consistent protein content, samples within one experiment were adjusted to the same total protein level by matching the lowest absorbance value.

Cell lysates were then mixed with 5× Laemmli buffer containing 10% glycerol (Sigma-Aldrich), 1% β-mercaptoethanol (Sigma-Aldrich), 1.7% SDS (Carl Roth), 62.5 mM Trizma base (pH 6.8; Sigma-Aldrich), and bromophenol blue (Sigma-Aldrich), followed by heating at 95°C for 5 min. Proteins were separated by sodium dodecyl sulfate polyacrylamide gel electrophoresis (SDS–PAGE) using self-cast 8%, 10%, or 12% acrylamide (Carl Roth) gels in a Mini-PROTEAN Tetra system (Bio-Rad) with running buffer (0.2 M glycine [Sigma-Aldrich], 25 mM Trizma base [Sigma-Aldrich], 0.1% SDS [Sigma-Aldrich]) at 80–120 V. Subsequently, proteins were transferred onto a polyvinylidene difluoride (PVDF) membrane (Merck Millipore) at 45 V for 2 h in 1x transfer buffer (0.1 M glycine, 50 mM Trizma base, 0.01% SDS, 10% methanol [Thermo Fisher Scientific], pH 8.3). Membranes were blocked for 1 h at RT in 3% skim milk powder (Gerbu) or 5% BSA (Carl Roth) in TBST (0.15 M NaCl [Sigma-Aldrich], 60 mM Trizma base, 3 mM KCl [Sigma-Aldrich], 0.1% Tween-20 [Sigma-Aldrich], pH 7.4).

Primary antibodies (Cell Signaling Technology: adme-R [#13522], sdme-RG [#13222], mme-R [#8051], eIF2α [#9722], eIF2α-pS51 [#9721]; Proteintech: TDO2 [#15880-1-AP]) were diluted as recommended by the manufacturer in 5% BSA/TBST containing 0.1% sodium azide (AppliChem) and incubated overnight at 4°C. The following day, membranes were washed three times for 10 min in TBST and then incubated for 2 h with the respective HRP-coupled secondary antibodies (Thermo Fisher Scientific) in 3% milk/TBST. After three additional 10 min washes in TBST, proteins were detected on a ChemiDoc XRS+ camera system (Bio-Rad) using ECL Western blotting Substrate (Thermo Fisher Scientific) or SuperSignal West FEMTO (Thermo Fisher Scientific) for low-abundance proteins. Images were quantified in Image Lab software (v6.0.1; Bio-Rad), and normalization was performed as described (Prentzell et al, 2021). Briefly, the pixel values of each lane were first normalized to the average of all lanes in a blot for each antibody, and the resulting values were normalized to tubulin ([EPR1333] #ab108629; Abcam).

### Nascent proteome analysis

**Figure S5.**
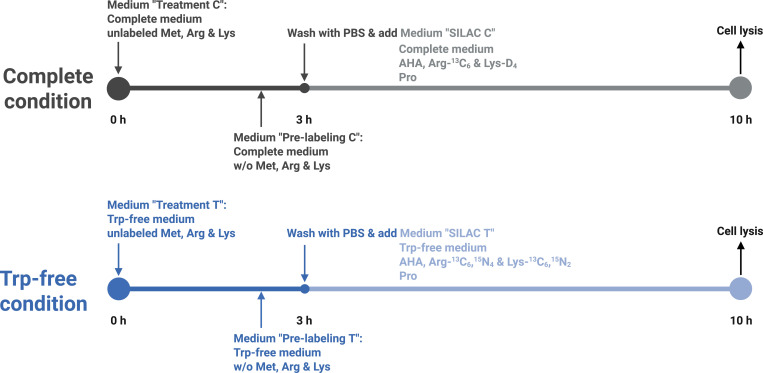
Scheme of cell culture media used for nascent proteome analysis.

To identify newly translated proteins upon Trp stress, Trp-depleted LN-229 were analyzed by a nascent-proteome approach as previously published (Fig 6F) (Eichelbaum et al, 2012; Borteçen et al, 2023). In brief, 3.8 × 10^6^ LN-229 cells were seeded per 10 cm cell culture dish (TPP) in complete DMEM and allowed to attach overnight. The next day, the medium was changed as described below to medium either containing or lacking 78 μM Trp for a total duration of 10 h (Fig S5). Double the number of dishes were seeded for the Trp-free condition as cells approximately halve their proliferation rate in the absence of Trp. All of the following self-made media for the nascent-proteome analysis contained 10% dialyzed FBS to prevent indirect re-supplementation of amino acids that could interfere with the nascent-proteome approach (Table S2).

For the first 3 h of the 10 h starvation period, complete or Trp-free culture medium (“Treatment C” or “Treatment T”, Table S2) contained unlabeled Met (201 μM; US biological), Arg (398 μM; Genaxxon) and Lys (798 μM; Sigma-Aldrich). To enhance labeling efficiency, “Treatment C” and “Treatment T” medium was replaced with complete or Trp-free medium (“Pre-labeling C” or “Pre-labeling T”), lacking Met, Arg and Lys. After 45 min, the “Pre-labeling C” or “Pre-labeling T” medium was again replaced with the respective SILAC labeling medium containing or lacking Trp (“SILAC C” or “SILAC T”). Both SILAC labeling media contained 100 μM of the clickable Met analog 4-Azido-*L*-homoalanine (AHA, Jena Biosciences) for pulse labeling of newly translated proteins and intermediate or heavy SILAC amino acids (“SILAC C”: intermediate, Arg-^13^C_6_ and Lys-D_4_; “SILAC T”: “heavy,” Arg-^13^C_6_,^15^N_4_ and Lys-^13^C_6_,^15^N_2_) to differentiate between the complete or Trp-free medium condition. Pulse labeling with “SILAC C” or “SILAC T” was performed for 7 h. Note: Because of AHA is known to be toxic for cells upon prolonged exposure, it is recommended to assess the tolerance of the individual cell type, both alone and in combination with the desired treatment. It is advisable to avoid phenol red-containing medium and tris(hydroxymethyl)-aminomethane (Tris)-containing buffers for the cell harvest as this may interfere with the click reaction. In addition, Proline (Pro, 0.2 g/liter or 1.74 mM; Genaxxon) was added throughout the last 7 h together with AHA and the SILAC amino acids to prevent Arg-to-Pro conversion (Bendall et al, 2008).

After 10 h, cells were washed with ice-cold PBS twice and collected by scraping with a rubber spatula. Cell pellets were frozen in liquid nitrogen and stored at −80°C until further processing. Newly synthesized, AHA-labeled proteins were enriched using click chemistry via a semi-automated protocol and subsequently analyzed by LC-MS/MS as already published (Borteçen et al, 2023).

More specifically, magnetic alkyne agarose (MAA) beads were prepared by coupling 1 M propargylamine (SantaCruz Biotechnology) to epoxy-activated magnetic agarose beads (Cube Biotech) for 16 h at 45°C. Cells pellets were lysed using a lysis buffer containing 1% SDS, 300 mM HEPES (pH 8.0) and complete EDTA-free protease inhibitor cocktail (Roche). Lysates were sonicated with a probe sonicator (Branson) at 10% power for 1 min. Protein concentrations were determined via Pierce BCA Protein Assay Kit. 200 μg protein (100 μg per condition) were diluted in 150 μl lysis buffer and used as input for the semi-automated enrichment protocol. To prevent the coupling of proteins containing strongly nucleophilic cysteine to the MAA beads (Ekkebus et al, 2013), the samples were alkylated by the addition of 3.4 μl of 600 mM iodoacetamide (IAA) (Bio-Rad) for 20 min at RT. Subsequently, 20 μl of MAA beads (Cytiva), diluted in lysis buffer were added to the samples. Newly synthesized proteins were covalently bound to MAA beads via copper(I)-catalyzed azide alkyne cycloaddition (CuAAC), which was initiated via addition of reaction mixture containing 21.62 mM copper sulfate (CuSO_4_, Sigma-Aldrich), 108.11 mM Tris-hydroxypropyltriazolylmethylamine (THPTA, Sigma-Aldrich), 216.22 mM pimagedine hydrochloride (aminoguanidine hydrochloride, Sigma-Aldrich) and 216.22 mM sodium ascorbate (Sigma-Aldrich). The sample plate was removed from the liquid handling platform, sealed using VersiCap Mat 96-well flat cap strips (Thermo Fisher Scientific) and incubated for 2 h at 40°C in a thermal shaker at 2.41*g*. The supernatant was removed on the liquid handling platform and MAA beads were washed with 150 μl milliQ water. Proteins bound to MAA beads were subsequently reduced and alkylated by the addition of 150 μl of 10 mM Tris(2-carboxylethyl)phosphine (TCEP) (Sigma-Aldrich) and 40 mM 2-chloroacetamide (CAA) (Sigma-Aldrich), dissolved in 100 mM Tris–HCl buffer (pH 8.0), containing 200 mM NaCl, 0.8 mM EDTA (Sigma-Aldrich), and 0.8% SDS. MAA beads were then incubated on the heating station at 70°C for 20 min and subsequently at 20°C for 15 min on the orbital shaker. MAA beads were subsequently washed three times with 1% SDS in 100 mM Tris–HCl (pH 8.0), 250 mM NaCl and 1 mM EDTA buffer, once with milliQ H_2_O, three times with 6 M guanidine-HCl (Sigma-Aldrich) in 100 mM Tris–HCl (pH 8.0) and three times with 70% ethanol, in consecutive washing steps of 150 μl each. Following washing, MAA beads were resuspended in 50 μl 100 mM ammonium bicarbonate buffer (pH 8.0). Proteins were digested off the MAA beads by adding 6 μl of 1 μg/μl sequencing grade trypsin (Promega), diluted in 50 mM acetic acid, for 16 h at 37°C in a thermal shaker. Tryptic peptides were lyophilized using a UNIVAPO-150H vacuum concentrator, coupled to a UNICRYO MC2 cooling trap and UNITHERM 4/14 D closed circuit cooler (UNIEQUIP) and subsequently purified using a modified SP3 peptide purification protocol (Hughes et al, 2014).

Magnetic carboxylate Sera-Mag SpeedBeads (Cytiva) were diluted to a concentration of 100 μg/μl in 10% FA and 5 μl were added to each lyophilized sample. Aggregation of the peptides onto beads was induced via the addition of 195 μl acetonitrile and incubating for 18 min while shaking at 0.017*g*. Next, the supernatant was removed and the Sera-Mag beads were washed two times with 180 μl acetonitrile and subsequently air-dried. The Sera-Mag beads were resuspended in 20 μl 0.1% FA in water and sonicated in an Ultrasonic Cleaner USC-T (VWR) for 10 min, and the supernatant was transferred to a new plate. The purified peptides were diluted in 0.1% FA and used for LC-MS/MS analysis.

LC-MS/MS measurements of the nascent-proteome samples were carried out using an EASY-nLC 1200 system (Thermo Fisher Scientific) coupled to an Orbitrap Fusion Tribrid mass spectrometer (Thermo Fisher Scientific).

Peptides were separated by reverse-phase liquid chromatography using 0.1% FA (solvent A) and 80% acetonitrile (solvent B) as mobile phases. Peptide separation occurred on an Acclaim PepMap trap column (Thermo Fisher Scientific, C18, 20 mm × 100 μm, 5 μm C18 particles, 100 Å pore size) and a nanoEase M/Z peptide BEH C18 analytical column (Waters, 250 mm × 75 μm 1/PK, 130 Å, 1.7 μm). Samples were loaded onto the trap column with the constant flow of solvent A at a maximum pressure of 800 bar. The analytical column was equilibrated with 2 μl solvent A at a maximum pressure of 600 bar heated to 55°C using a HotSleeve+ column oven (Analytical SALES and SERVICES). The peptides were eluted with a constant flow rate of 300 nl/min. The concentration of solvent B was gradually increased during the elution of the peptides. The gradient started with 4% solvent B and was increased to 6% solvent B in the first 1 min, increased to 27% solvent B at 70 min and further increased to 44% solvent B after 85 min. After 85 min, the percentage of solvent B was raised to 95%. After 95 min, the system was re-equilibrated using 5% solvent B for 10 min. Eluting peptides were ionized and injected into the mass spectrometer, using the Nanospray flex ion source (Thermo Fisher Scientific) and a Sharp Singularity nESI emitter (ID = 20 μm, OD = 365 μm, L = 7 cm, α = 7.5°) (FOSSILIONTECH), connected to a SIMPLE LINK UNO-32 (FOSSILIONTECH). A static spray voltage of 2.5 kV was applied to the emitter and the capillary temperature of the ion transfer tube was set to 275°C. The Orbitrap Fusion Tribrid mass spectrometer was operated in data-dependent mode with a full scan range of 375–1,500 *m/z*, Orbitrap resolution of 60,000 FWHM, automatic gain control (AGC) target of 3 × 10^6^ and maximum injection time of 32 ms was set and MS/MS spectra were acquired in the linear ion trap operated in “Rapid” mode with a fixed cycle time of 3 s. MS/MS scan range was set from 200 to 2,000 *m/z*, dynamic exclusion was set to 30 s and monoisotopic peptide precursor selection was enabled.

Raw files were processed using MaxQuant (version 2.0.3) (Cox & Mann, 2008) and the Andromeda search engine (Cox et al, 2011), using a human proteome fasta file, retrieved from the SwissProt database (version from February 2021 with 20,934 entries). The enzymatic digestion was set to Trypsin/P and a maximum of two missed cleavages per peptide were allowed.

In this SILAC experiment, two differentially labeled cell populations were prepared to enable simultaneous quantitative comparison of protein expression. The intermediate channel, referred to as SILAC C, used Arg labeled with six carbon-13 atoms (Arg-^13^C_6_ also known as Arg6) and Lys labeled with four deuterium atoms (Lys-D_4_, also known as Lys4), resulting in a moderate mass shift in peptides. The heavy channel, SILAC T, incorporated heavy-labeled amino acids: Arg with six carbon-13 and four nitrogen-15 atoms (Arg-^13^C_6,_^15^N_4_, or Arg10) and Lys with six carbon-13 and two nitrogen-15 atoms (Lys-^13^C_6_,^15^N_2_, or Lys8), causing a larger mass shift. Cysteine carbamidomethylation was set as fixed modification, whereas Met oxidation, N-terminal acetylation, and deamidation of asparagine and glutamine were set as variable peptide modifications. Minimum peptide length was set to seven and max. peptide mass was set to 4,600 Da. PSM-, protein-, and site decoy fraction FDR were set to 1%. The minimum delta score threshold for unmodified peptides was set to 6, and 40 for modified peptides. Unique and razor peptides were used for quantification and normalized SILAC ratios and iBAQ values were calculated. Minimum ratio count was set to 0 to not exclude identifications in single SILAC channels. The re-quantify and match-between-runs were enabled and other settings were left with default parameters. Differential protein abundance analysis was carried out using the *limma* ([version 3.46.0] [Ritchie et al, 2015]) and DEqMS ([version 1.8.0] [Zhu et al, 2020]) R/Bioconductor packages, by fitting the data onto a linear model and performing an empirical Bayes moderated *t* test. The number of precursors, with consideration of modified peptide sequences and charge but not SILAC channels, of each protein group was included as a factor for the variance estimation in DEqMS. SILAC ratios were used to quantify relative changes in the nascent proteomes, shown as log_2_ fold change (log_2_ FC) in a volcano plot. Significantly up-regulated proteins are indicated in light grey (−log_10_ *P*-value > 1.301).

Nascent-proteome data have been uploaded to the PRIDE repository (Perez-Riverol et al, 2022) and can be accessed with the Project Accession Number PXD067012.

### Statistical analysis

Statistical analyses and graphical presentation were conducted using GraphPad Prism (version 9.4.1). Data are presented as mean ± SEM. For pairwise comparisons, either a paired (immunoblots) or unpaired (metabolite measurements) two-tailed *t* test was applied. If more than two conditions were compared either a one-way (Seahorse measurements, microscopy images) or two-way ANOVA (SRB assay) with a Šidák’s multiple comparisons test was applied. The number of biological replicates and the specific statistical test used are mentioned in each figure legend. Asterisks indicate levels of statistical significance with *P < 0.05, **P < 0.01, ***P < 0.001 and ****P < 0.0001. ns = *P* > 0.05 is not depicted in the figures itself, but mentioned in the respective figure legends.

## Supplementary Material

Reviewer comments

## Data Availability

Nascent-proteome data have been uploaded to the PRIDE repository and can be accessed with the Project Accession Number PXD067012. Further information and requests for resources and reagents should be directed to the corresponding authors MT Prentzell (t.prentzell@dkfz.de) and CA Opitz (c.opitz@dkfz.de).
