# Supplementary material for: A step-by-step guide to performing cancer metabolism research using custom-made media
Source: Life Sci Alliance. 2025 Dec 10;9(2):e202503529. doi: 10.26508/lsa.202503529 (PMC12696398; doi:10.26508/lsa.202503529)
Supplement: Supplementary file 2 [file LSA-2025-03529_TableS1.docx]

**Structured Methods - Reagents and Tools Table**

| **Reagent/Resource** | **Reference or Source** | **Identifier or Catalog Number** |
| --- | --- | --- |
| **Experimental Models** | | |
| A-172 (*H. sapiens*) | American Type Cell Culture Collection (ATCC) | CRL-1620 |
| HeLa S3 (*H. sapiens*) | ATCC | CCL-2.2 |
| LN-229 (*H. sapiens*) | ATCC | CRL-2611 |
| NCE-G 142 (*H. sapiens*) | Kind donation from the laboratory of Dr. Katrin Lamszus (University Hospital Hamburg-Eppendorf) |  |
| **Antibodies** | | |
| adme-R | Cell Signaling Technology | 13522 |
| eIF2α | Cell Signaling Technology | 9722 |
| eIF2α-pS51 | Cell Signaling Technology | 9721 |
| Goat anti-rabbit IgG | Thermo Fisher Scientific | 31460 |
| mme-R | Cell Signaling Technology | 8051 |
| sdme-RG | Cell Signaling Technology | 13222 |
| TDO2 | Proteintech | 15880-1-AP |
| Tubulin [EPR1333] | Abcam | ab108629 |
| **Chemicals, Enzymes and other reagents** | | |
| 2-Chloroacetamide (CAA) | Sigma Aldrich | C0267 |
| β-Mercaptoethanol | Sigma Aldrich | M3148 |
| Acetic acid, glacial | Carl Roth | 37384 |
| Ammonium acetate | Sigma Aldrich | 73594 |
| Ammonium bicarbonate | Sigma Aldrich | A6141 |
| Bovine serum albumin | Carl Roth | 8076.3 |
| Bromophenol blue | Sigma Aldrich | B5525 |
| Chloroform | Sigma Aldrich | 650498 |
| Complete Protease Inhibitor Cocktail | Roche | 11836145001 |
| Complete EDTA-free Protease Inhibitor Cocktail | Roche | 11873580001 |
| Copper sulfate (CuSO_4_) | Sigma Aldrich | 451657 |
| Cytiva Sera-Mag SpeedBeads™ Carboxyl-Magnet-Beads, hydrophilic | Cytiva | 11548692 |
| Cytiva Sera-Mag SpeedBeads™ Carboxyl-Magnet-Beads, hydrophobic | Cytiva | 11819912 |
| Ethylendiaminetetraacetic acid (EDTA) | Sigma Aldrich | COMH9A1B755F |
| Formic acid | Biosolve | 6914143 |
| Glycerol | Sigma Aldrich | 15523 |
| Guanidine-HCl | Sigma Aldrich | G3272 |
| HEPES sodium salt | Sigma Life Science | H7006 |
| Hydrochloric acid (HCl), 25% | Carl Roth | X897.1 |
| IGEPAL | Sigma Aldrich | I8896 |
| Iodoacetamide (IAA) | Bio-Rad | #1632109 |
| Isopropanol | Thermo Fisher Scientific | P/7500/PC17 |
| LC-MS grade acetonitrile | Sigma Aldrich/Merck Millipore | 900667/100029 |
| LC-MS grade methanol | VWR/Merck Millipore | 20.864.320/106035 |
| LC-MS grade water | Merck Millipore | 115333 |
| Methanol | Thermo Fisher Scientific | M/4000/PC17 |
| PageRuler™ Plus Prestained Protein Ladder, 10-250 kDa | Thermo Fisher Scientific | 26620 |
| Phosphatase Inhibitor Cocktail 2 | Sigma Aldrich | P5726 |
| Phosphatase Inhibitor Cocktail 3 | Sigma Aldrich | P0044 |
| Pierce^TM^ BCA Protein Assay Kit | Thermo Fisher Scientific | 23227 |
| Pierce™ ECL Western Blotting Substrate | Thermo Fisher Scientific | 32106 |
| Pimagedine hydrochloride (aminoguanidine hydrochloride) | Sigma Aldrich | 396494 |
| Precision Plus Protein™ Dual Color Standards | Bio-Rad | 1610394 |
| Propargylamine | SantaCruz Biotechnology | sc-253321 |
| Protein Assay Dye Reagent Concentrate | Bio-Rad | 5000006 |
| PureCube Epoxy Activated MagBeads | Cube Biotech | 50805 |
| ROTIPHORESE®Gel 30 (Acrylamide) | Carl Roth | 3029.1 |
| Sequencing grade Trypsin | Promega | V5111 |
| Skim milk powder | Gerbu | 16020500 |
| Sodium ascorbate | Sigma Aldrich | A7631 |
| Sodium azide | AppliChem | A1430,0100 |
| Sodium deoxycholate | AppliChem | A1531 |
| Sodium dodecyl sulfate (SDS) | Carl Roth | 8029.3 |
| Sodium hydroxide (NaOH) | Reagecon | 3006300 |
| Sulforhodamine B (SRB) sodium salt | Sigma Aldrich | S1402 |
| SuperSignal™ West Femto Maximum Sensitivity Substrate | Thermo Fisher Scientific | 34096 |
| Trichloroacetic acid (TCA) | Carl Roth | 7437.1 |
| Tris(2-carboxyethyl)phosphine hydrochloride (TCEP) | Sigma Aldrich | C4706 |
| Tris(3-hydroxypropyltriazolylmethyl)amine (THPTA) | Sigma Aldrich | 762342 |
| Tris(hydroxymethyl)-aminomethane-HCl | Carl Roth | 9090.3 |
| Trizma® base | Sigma Aldrich | T1503 |
| Tween-20 | Sigma Aldrich | P9416 |
| **Reagents for cell culture** | | |
| Antimycin A | Santa Cruz | 202467 |
| Cell culture grade water | Corning | 25-055-CV |
| *D*-(+)-Glucose solution | Sigma Aldrich | G8769 |
| *D*-Glucose-^13^C_6_ | Cambridge Isotope Laboratories Inc. | CLM-1396-PK |
| Dialyzed Fetal Bovine Serum (DFBS) | Gibco®, Thermo Fisher Scientific | 26400 |
| DMEM w/ 1.0 g/L glucose, w/o glutamine, w/o amino acids | Genaxxon | C4150.0500 |
| Dulbecco's Modified Eagle Medium (DMEM) w/ 4.5 g/L glucose | Gibco®, Thermo Fisher Scientific | 31053-028 |
| Fetal Bovine Serum (FBS) | Gibco®, Thermo Fisher Scientific | 26140079 |
| Mycoplasma test kit Venor®GeM Classic | Minerva Biolabs GmbH | 11-1025 |
| Oligomycin A | Cayman Chemicals | 11342 |
| Penicillin-Streptomycin | Thermo Fisher Scientific | 15140122 |
| Phosphate buffered saline (PBS) | Gibco®, Thermo Fisher Scientific | 14190094 |
| Rotenone | Sigma Aldrich | R8875-1G |
| Sodium pyruvate | Gibco®, Thermo Fisher Scientific | 11360 |
| Trifluoromethoxy carbonylcyanide phenylhydrazone (FCCP) | Cayman Chemicals | 15218 |
| Trypan blue stain (0.4%) | Invitrogen | T10282 |
| Trypsin w/ 5% EDTA | Gibco®, Thermo Fisher Scientific | 15400054 |
| **Amino acids** | | |
| 4-Azido-*L*-homoalanine HCl (AHA) | Jena Biosciences | CLK-AA005 |
| Glycine | Sigma Aldrich | G8790 |
| *L*-Arginine (Arg) base | Genaxxon | M6086.0100 |
| *L*-Arginine-^13^C_6_ (Arg-^13^C_6_) | Silantes | 201204102 |
| *L*-Arginine-^13^C_6_,^15^N_4_ (Arg-^13^C_6_,^15^N_4_) | Silantes | 201604102 |
| *L*-Cystine dihydrochloride | Sigma Aldrich | C2526 |
| *L*-Glutamine | Gibco®, Thermo Fisher Scientific | 25030-024 |
| *L*-Histidine hydrochloride monohydrate | Genaxxon | M6109.0100 |
| *L*-Isoleucine | Genaxxon | M6113.0100 |
| *L*-Leucine | Sigma Aldrich | 61819 |
| *L*-Lysine monohydrochloride (Lys) | Sigma Aldrich | L8662 |
| *L*-Lysine-^13^C_6_,^15^N_2_ (Lys-^13^C_6_,^15^N_2_) | Silantes | 211604102 |
| *L*-Lysine-D_4_ (Lys-D_4_) | Silantes | 211104113 |
| *L*-Methionine (Met) | US biological | M3020 |
| *L-*Methionine*-*^13^C_5_,^15^N | Sigma Aldrich | 608106 |
| *L*-Phenylalanine | Carl Roth | 1709.1 |
| *L*-Proline (Pro) | Genaxxon | M6126.0050 |
| *L*-Serine | Genaxxon | M6128.0025 |
| *L*-Threonine | Sigma Aldrich | 89179 |
| *L*-Tryptophan (Trp) | Sigma Aldrich | T8941 |
| *L*-Tryptophan-^13^C_11_,^15^N_2_ (Trp-^13^C_11_,^15^N_2_) | Sigma Aldrich | 574597 |
| *L*-Tyrosin disodium salt dihydrate | Thermo Fisher Scientific | J61770.22 |
| *L*-Valine | Genaxxon | M6136.0025 |
| **Vitamins** | | |
| ^18^O-Nicotinamide (^18^O-NAM) | Marie Migaud Laboratory | synthesized as described previously in Makarov et al, 2019 |
| Choline chloride | Sigma Aldrich | C7527 |
| *D*-Calcium pantothenate | Sigma Aldrich | 21210 |
| Folic acid | Sigma Aldrich | F7876 |
| *i*-Inositol | Sigma Aldrich | I7508 |
| Nicotinamide (NAM) | Sigma Aldrich | 72340 |
| Pyridoxine hydrochloride | Sigma Aldrich | P6280 |
| Riboflavin | Sigma Aldrich | R9504 |
| Thiamine hydrochloride | Sigma Aldrich | T1270 |
| **Inorganic salts** | | |
| Calcium chloride dihydrate | Sigma Aldrich | 21097 |
| Iron(III) nitrate nonahydrate | Sigma Aldrich | F8508 |
| Magnesium sulfate | AppliChem | A4101 (131404) |
| Potassium chloride | Sigma Aldrich | 67811 (P3911) |
| Sodium bicarbonate | Sigma Aldrich | S5761 |
| Sodium chloride | Sigma Aldrich | 31434 |
| Sodium dihydrogen phosphate dihydrate | Sigma Aldrich | 1.06345 |
| **Consumables** | | |
| 0.5 mL Safe-Lock Tubes | Eppendorf | 0030121.023 |
| 1.5 mL Safe-Lock Tubes | Eppendorf | 0030120.086 |
| 2 mL Safe-Lock Tubes | Eppendorf | 0030120.094 |
| 5 mL serological pipette | Thermo Fisher Scientific | 15082 |
| 6-well cell culture plate | Greiner Bio-One | 657160 |
| 12-well cell culture plate | TPP | 92412 |
| 6 cm cell culture dish | Greiner Bio-One | 628160 |
| 10 cm cell culture dish | TPP | 93100 |
| 15 cm cell culture dish | TPP | 93150 |
| 10 mL serological pipette | Greiner Bio-One | 607180 |
| 25 mL serological pipette | Greiner Bio-One | 760180 |
| 15 mL conical tube | Greiner Bio-One | 188271-N |
| 50 mL conical tube | Greiner Bio-One | 227261 |
| µ-Plate 24 Well | ibidi GmbH | 82426 |
| Combitips® advanced 10 mL | Eppendorf | 003089.456 |
| Combitips® advanced 5 mL | Eppendorf | 0030089.464 |
| Countess™ Cell Counting Chamber Slides | Invitrogen | C10312 |
| HPLC glass vials and caps | Agilent | 8010-0542 |
| Microplate 96-well, F-bottom, black | Greiner Bio-One | 655900 |
| Microplate 96-well, F-bottom, clear | Greiner Bio-One | 655101 |
| Polyvinylidene difluoride (PVDF) membranes for immunoblotting (Pore size 0.45 µM) | Merck Millipore | IPVH00010 |
| Stericup® Quick Release Vacuum Driven Sterile Filter 500 mL, 0.22 µm pore size | Merck Millipore | S2GPU05RE |
| T175 cell culture flask | Greiner Bio-One | 159900 |
| T75 cell culture flask | Greiner Bio-One | 658170 |
| TipOne® 10/20 µL XL graduated filter tip | Starlab | S1120-3710 |
| TipOne® 10/20 µL XL graduated tip | Starlab | S1111-3700 |
| TipOne® 1000 µL blue graduated tip | Starlab | S1111-6701 |
| TipOne® 1000 µL XL graduated filter tip | Starlab | S1122-1730 |
| TipOne® 20 µL bevelled filter tip | Starlab | S1120-1710 |
| TipOne® 200 µL graduated filter tip | Starlab | S1120-8710 |
| TipOne® 200 µL yellow tip | Starlab | S1111-0706 |
| Tissue culture test plate 24-well | Greiner Bio-One | 662160 |
| VersiCap Mat 96-well flat cap strips | Thermo Fisher Scientific | AB1815 |
| Whatman Grade 3MM Chr Blotting Paper | Cytiva | 3030-917 |
| **Instruments and laboratory equipment** | | |
| Acclaim PepMap trap column, C18, 20 mm × 100 μm, 5 μm C18 particles, 100 Å pore size | Thermo Fisher Scientific |  |
| ACQUITY Premier HSS T3 Column with VanGuard FIT, 1.8 µm, 2.1 x 150 mm | Waters^TM^ | SKU:186009472 |
| ACQUITY Premier HSS T3 VanGuard FIT Cartridge, 1.8 µm, 2.1 x 5 mm | Waters^TM^ | SKU:186009473 |
| Acquity Waters Premier System | Waters^TM^ |  |
| Analytical balance | Kern & Sohn | ABJ 320-4NM |
| Atlantis Premier BEH Z-HILIC 2.5 µm VanGuard FIT 2.1 x 100 mm | Waters^TM^ |  |
| Bravo Automated Liquid Handling Platform | Agilent |  |
| Cell culture incubator | SANYO Electric Co., Ltd. | MCO-20AIC CO2 Incubator |
| Centrifuge | Eppendorf | 5810 R |
| ChemiDoc XRS+ camera system | Bio-Rad | 1708265 |
| CLARIOstar® microplate reader | BMG Labtech |  |
| Dionex UltiMate 3000 liquid chromatography system (UPLC) | Thermo Fisher Scientific |  |
| EASY-nLC 1200 | Thermo Fisher Scientific |  |
| Heraeus™ B12 Function Line Incubator | Thermo Fisher Scientific |  |
| HotSleeve+ column oven | Analytical SALES & SERVICES |  |
| MICA Microhub | Leica® |  |
| Mini-PROTEAN Tetra Vertical Electrophoresis Cell System | Bio-Rad | 1658029FC |
| Multichannel pipette (0.5-10 µl) | Socrorex | 855.08.010 |
| Multipette | Eppendorf | 4987000010 |
| nanoEase M/Z peptide BEH C18 analytical column (250 mm × 75 μm, 130 Å, 1.7 μm) | Waters^TM^ | 186008795 |
| Nanospray flex ion source | Thermo Fisher Scientific |  |
| Orbitrap Fusion Tribrid mass spectrometer | Thermo Fisher Scientific |  |
| pH meter | inoLab | WTW™ 7110/1AA114 |
| Probe sonicator | Branson |  |
| Q Exactive Orbitrap Mass Spectrometer | Thermo Fisher Scientific |  |
| Seahorse FluxPaks | Agilent | 103793-100 |
| Seahorse XFe96 Analyzer | Agilent |  |
| Sharp Singularity nESI emitter | FOSSILIONTECH |  |
| SIMPLE LINK UNO-32 | FOSSILIONTECH |  |
| SpeedVac | Thermo Fisher Scientific |  |
| ThermoMixer® comfort | Eppendorf |  |
| Truview total recovery glass vials, Max Recovery | Waters^TM^ | 186005662CV |
| Ultrasonic bath “Digital 10 P” | Sonorex |  |
| UNICRYO MC2 cooling trap | UNIEQUIP |  |
| UNITHERM 4/14 D closed circuit cooler | UNIEQUIP |  |
| UNIVAPO-150H vacuum concentrator | UNIEQUIP |  |
| Vanquish Flex UPLC system | ThermoFisher Scientific |  |
| Water Bath “1005” | Gesellschaft Fuer Labortec™ |  |
| Zeno-TOF^TM^ 7600 System | AB SCIEX Germany GmbH |  |
| ZIC pHILIC column | Merck Millipore | 150460 |
| **Software** | | |
| CLARIOstar® Reader Control Software | BMG Labtech  https://www.bmglabtech.com/de/microplate-reader-software/ | V5.40 R2 |
| DEqMS | https://pubmed.ncbi.nlm.nih.gov/32205417/ | v.1.8.0 |
| GraphPad Prism | GraphPad Software Inc.  https://www.graphpad.com/scientific-software/prism/ | v.9.4.1 |
| Image Lab software | Bio-Rad  https://www.bio-rad.com/de-de/product/image-lab-software?ID=KRE6P5E8Z | v.6.0.1 |
| *limma* | http://www.ncbi.nlm.nih.gov/entrez/query.fcgi?cmd=Retrieve&db=PubMed&dopt=Abstract&list_uids=25605792 | v.3.46.0 |
| MARS Data Analysis Software | BMG Labtech  https://www.bmglabtech.com/de/microplate-reader-software/ | 3.31 |
| MaxQuant | Max Planck Institute of Biochemistry  https://www.maxquant.org/ | Version 2.0.3 |
| Metabolite AutoPlotter 2.3 | https://mpietzke.shinyapps.io/AutoPlotter/ | 2.6 |
| Open Microscopy Environment Remote Objects (OMERO) | The Open Microscopy Environment  https://omero-prod.cloud-app.dkfz.de/webclient/login/?url=%2Fwebclient%2F | 5.8.3 |
| Sciex OS | AB SCIEX Germany GmbH  https://sciex.com/support/software-support/software-downloads | v.3.3.0 |
| Seahorse XF Wave Analyzer | Agilent  https://www.agilent.com/en/product/cell-analysis/real-time-cell-metabolic-analysis/xf-software/seahorse-wave-controller-software-2-6-1-740904 | v. 2.6.1 |
| TraceFinder^TM^ Software | ThermoFisher Scientific https://www.thermofisher.com/order/catalog/product/OPTON-31001 | Version 4.1 |
| Xcalibur™ Software | ThermoFisher Scientific  https://www.thermofisher.com/order/catalog/product/OPTON-30965 | Version 4.1 |
